# Profiling Selectivity for the *Shigella* Virulence Factor OspF

**DOI:** 10.1021/acs.biochem.6c00109

**Published:** 2026-04-22

**Authors:** Nicholas P. McCurtin, Ariana Gazaferi, Noah D. Novick, Kyungsub Kim, Cammie F. Lesser, Rebecca A. Scheck

**Affiliations:** † Department of Chemistry, School of Arts and Sciences, Tufts University, 62 Talbot Avenue, Medford, Massachusetts 02155, United States; ‡ Department of Molecular Biology and Microbiology, Tufts University School of Medicine, 150 Harrison Avenue, Boston, Massachusetts 02111, United States; § Levy Center for Integrated Management of Antimicrobial Resistance, South Cove 502, 136 Harrison Ave, Boston, Massachusetts 02111, United States

## Abstract

Phosphothreonine lyases are a small family of bacterial virulence factors that are secreted into host cells during infection by some Gram-negative pathogens. Phosphothreonine lyases catalyze an irreversible phosphate β-elimination that converts phosphothreonine (pThr) to dehydrobutyrine (Dhb) on host proteins. Here, we explore the substrate profile for OspF, a phosphothreonine lyase secreted during *Shigella flexneri* infection, which has been previously reported to modify mitogen-activated protein kinases (MAPKs) to silence the host immune response. In this work, we use a combination of *in vitro* assays with synthetic phosphopeptides and bottom-up chemoproteomic profiling to uncover the OspF substrates. These studies not only show that OspF exhibits selectivity within the MAPK family but also reveal that OspF modifies a wide range of cellular targets in cellular lysates and during *Shigella flexneri* infection. Using a nucleophilic phosphine chemical probe, we identified and validated new OspF targets beyond the MAPK family, including Rab1A and casein kinase 2β. Together, these findings provide valuable insights about OspF substrates in *Shigella flexneri* infection and highlight the need for further studies to reveal its full role in the context of *Shigella flexneri* pathogenesis. Moreover, these data underscore the potential utility of OspF and other phospholyases as novel chemical and synthetic biology tools for site-specific protein editing.

## Graphical abstract

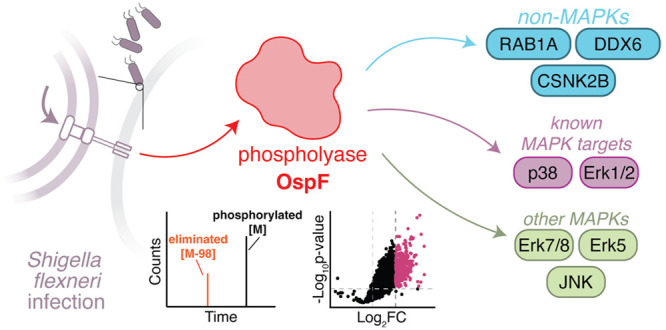

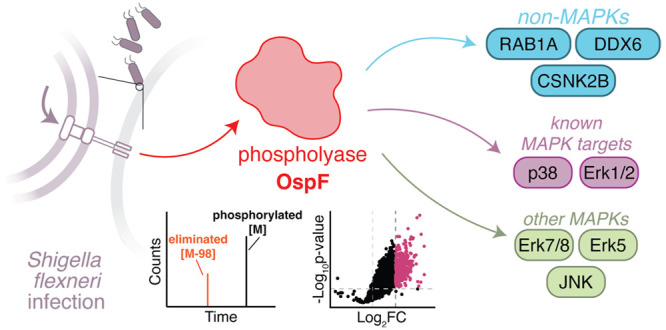

## Introduction

To evade host defense mechanisms, many pathogenic bacteria secrete virulence factors (or effectors) into the host cell, enabling them to subvert the host immune response.^1–7^ *Shigella flexneri* is one of a handful of pathogens that secrete a class of effectors known as phosphothreonine lyases, or phospholyases, that catalyze phosphate β-elimination from phosphothreonine (pThr) to generate dehydrobutyrine (Dhb) in its place (Figure 1).^8–13^ Dhb is thought to be a rare post-translational modification (PTM) that does not typically transpire in healthy mammalian cells and cannot be rephosphorylated by any known mammalian biochemical processes.^7,14^ As a result, exploiting phospholyase biochemistry could enable access to new chemical tools for disrupting phosphorylation networks, which may be especially beneficial both for revealing new aspects of phosphorylation-driven signaling and for a range of potential synthetic or chemical biology applications.

**Figure 1:**
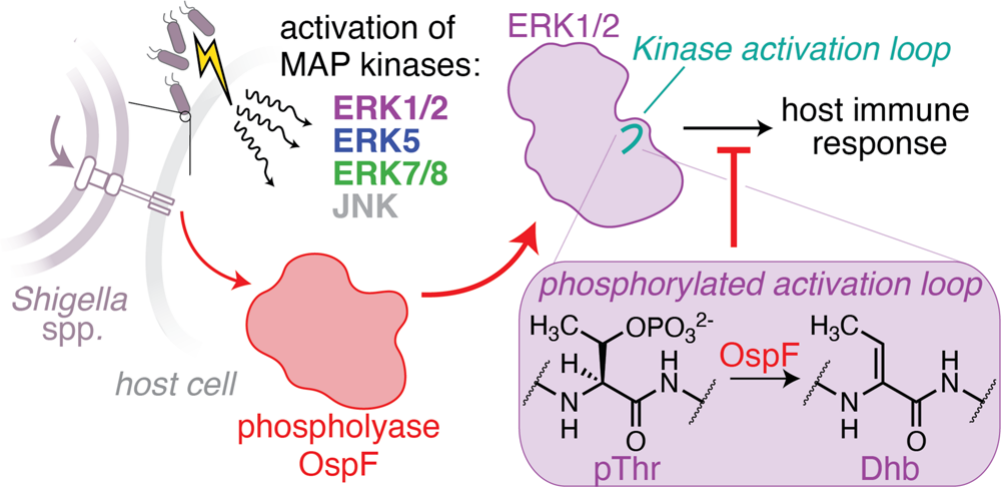
Upon infection, host machinery sets off a cascade of phosphorylation events to activate the immune system and fight off pathogens. OspF, an effector secreted from *Shigella flexneri*, catalyzes phosphate β-elimination on pThr residues to generate dehydrobutyrine (Dhb). This helps *Shigella* silence host phosphorylation and subvert the immune response.

The mitogen activated protein kinase (MAPK) signaling pathway is an essential hub for the host’s innate immune response. All known phospholyases possess a canonical docking domain (D-domain) that is conserved among MAPK-targeting proteins and are known to act on MAPKs, including the extracellular signal-regulated kinases 1/2 (ERK1/2) and p38. To date, it remains unknown whether phospholyases are highly specific for MAPK-related substrates or are instead broadly active against a range of host phosphorylated proteins. Recent work using synthetic phosphopeptide substrates^14^ has suggested that OspF exhibits a broader substrate scope than is currently appreciated. Furthermore, proteomic and gene expression studies have revealed widespread changes in cellular signaling due to *Shigella flexneri* infection, many of which were modulated in an OspF-dependent manner.^1,15^ However, these studies were unable to clarify if these are direct or downstream consequences of OspF activity.

Progress in revealing OspF function has been hampered by a lack of tools that can track and label phosphate β-elimination. Notably, unbiased proteomic analysis of OspF activity is extremely difficult due to the identical mass changes obtained for neutral loss of water and OspF-mediated phosphate β-elimination.^16–22^ To address this challenge, our lab recently developed a nucleophilic phosphine chemical probe that can selectively label Dhb, the major OspF product.^23^ In this work, we use this probe in a bottom-up chemoproteomic workflow alongside *in vitro* assays with synthetic phosphopeptides to evaluate OspF substrate preferences. These studies reveal nuanced aspects of the selectivity of OspF, showing it to be active against a broad range of substrates and also exhibits selectivity for specific substrates within the MAPK family. Together, these data underscore the potential utility of OspF, and phospholyases in general, for synthetic and chemical biology applications, while also highlighting the need for further studies to reveal its full role in the context of *Shigella flexneri* pathogenesis.

## Methods

### General

All chemical reagents were of analytical grade, obtained from commercial suppliers, and used without further purification unless otherwise noted. Water used in biological procedures was distilled and deionized using an Arium pro purification system (Sartorius). All statistical analysis was conducted using Prism GraphPad.

### Peptide Synthesis

Amidated peptides were synthesized on a Rink Amide MHBA resin on a 0.05 mmol scale following standard Fmoc peptide synthesis procedures in fritted syringes. Fmoc was deprotected using 20% piperidine in dimethylformamide (DMF). For more sensitive peptides, a 2% solution of 1,8-diazabicyclo[5.4.0]­undec-7-ene (DBU) in DMF was used to deprotect Fmoc to avoid base-catalyzed elimination. Fmoc-protection was accomplished by two iterative washes with one full syringe volume of DBU for 5 min at room temperature, followed by four iterative washes with one full syringe volume of DMF. For coupling, 5 equiv of amino acid was added to 5 equiv of *O*-(benzotriazol-1-yl)-*N*,*N*,*N*′,*N*′-tetramethyluronium hexafluorophosphate (HBTU), 5 equiv of hydroxybenzotriazole (HOBt), and 10 equiv of *N*,*N*-diisopropylethyllamine (DIPEA) in DMF and the mixture was allowed to preactivate for 5 min. The coupling solution was added to the fritted syringe and allowed to couple for 50 min at room temperature. For phosphorylated amino acids, coupling was extended to 1.5 h. After the final deprotection step, peptides were washed twice with one syringe volume of dichloromethane (DCM) for 15 min at room temperature and placed in a vacuum desiccator for 30 min. Peptides were resuspended in a cleavage cocktail (95% trifluoroacetic acid (TFA), 2.5% triisopropyl silane, 2.5% water) and incubated at room temperature for 3 h. The resulting peptide solution was concentrated under nitrogen and redissolved in a water/acetonitrile mixture before purification by RP-HPLC.

### Peptide Purification

Peptides were purified using an Agilent 1260 LC system equipped with an Agilent ZORBAX SB-C18 column (9.4 × 250 mm), 5 μm particle size employing water (A) and acetonitrile (B) mobile phase with 0.1% TFA. Fractions were characterized using matrix-assisted laser desorption/ionization time-of-flight (MALDI-TOF) mass spectrometry (Bruker) and an Agilent 6530 quadrupole time-of-flight (Q-TOF) mass spectrometer coupled to an Agilent 1260 HPLC system. Pure fractions were combined, lyophilized, and stored at −20 °C as a 10 mM stock solution in water.

### Generation of Mutant Enzymes

Point-mutated OspF genes were produced using a QuikChange II Site-Directed Mutagenesis Kit (Agilent). Primers were designed using an Agilent QuickChange Primer Design Tool and purchased from Invitrogen ThermoFisher. Two μL of the PCR reaction was transformed into XL10-Gold ultracompetent (Agilent) *E. coli*. The following day, colonies were inoculated into 5 mL of Luria Broth with 1:1000 ampicillin and grown at 37 °C overnight (16–18 h). Cultures were pelleted at 4,700 rpm for 15 min and then mini-prepped using the QIAprep Spin Miniprep Kit. Extracted DNA was sequenced by Azenta GeneWiz, and concentrations were determined using a Tecan Spark 10 M microplate reader. OspF mutants were transformed into BL21-CodonPlusRIL (Agilent) *E. coli* and expressed as described in this paper. For the ΔN26 mutant, the gene was designed and purchased from GeneArt, and then, the above steps were followed.

### Protein Expression and Purification

Phospholyases were produced as described previously.^14^ Briefly, an *Escherichia coli* optimized copy containing the OspF gene was cloned into the pRSETA vector, which fuses target proteins with an N-terminal His-tag. Expression vectors encoding the OspF were transformed into BL21-CodonPlusRIL (Agilent) *E. coli*. Cultures were grown to an OD_600_ of 0.4–0.6 at 37 °C, and expression was induced with 0.1 mM isopropyl β-d-1thiogalactopyranoside (IPTG) at 20 °C for 3–4 h. After expression, the cells were pelleted and frozen. BugBuster protein extraction reagent was used along with 0.5 mM phenylmethanesulfonyl fluoride and Benzonase nuclease to lyse bacterial cells. Proteins were purified using a His-GraviTrap protein purification column with elution buffer (500 mM imidazole, 100 mM Tris, and 50 mM NaCl (pH 7)). A buffer exchange was performed using Tris-buffered saline (100 mM Tris and 50 mM NaCl (pH 7)) to remove imidazole. The resulting pure protein samples were stored at −20 °C. Concentrations were determined by using a Bio-Rad Bradford Protein Assay and a Tecan Spark 10 M microplate reader.

### General Peptide Elimination Protocol

Synthetic phosphopeptide elimination studies were conducted by incubating 1 mM peptide in TBS at pH 8 at 26 °C. Reaction was initiated upon the addition of 10 μM recombinant enzyme. To quench reactions, 5 μL of the sample was diluted in 200 μL of 100 mM HCl.

### Peptide Study LC-MS Data Acquisition

Reversed-phase chromatography and mass spectrometry were performed on an Agilent 1260 Infinity LC system in line with an Agilent 6530 Accurate Mass Q-TOF. Reversed-phase chromatography was performed on a ZORBAX 30SB-C8 column (2.1 mm × 100 mm, Agilent) using a water/acetonitrile gradient mobile phase containing 0.1% formic acid (0.4 mL/min; 2% ACN, isocratic 0–1.75 min, 2–48%, 1.76–16 min). Data analysis was performed by using Agilent MassHunter Qualitative Analysis software. Conversion from the phosphorylated parent peak to the eliminated product peak was calculated based on the detected peak volumes. Peak volumes (*V*’s) are determined using the Molecular Feature Extractor within the Agilent Qualitative Analysis software and represent ion counts observed for any and all charge states associated with a particular parent ion as previously described.^14,23–25^ As peptides and their modified counterparts can ionize differently (e.g., different charge states or different salt adducts), this method provides a more robust measure than comparing only a single charge state. A list of all peptides used and their *m*/*z* values are found in Table S1.

1 $$\%\;apparent\;conversion=100\times \frac{V_{product}}{V_{product}+V_{parent}}$$

### Cell Culture

HeLa cells were purchased from ATCC (CCL-2) and grown in Dulbecco’s Modified Eagle Medium supplemented with 10% (v/v) Fetal Bovine Serum and 10% (v/v) Penicillin–Streptomycin at 37 °C with 5% CO_2_. Cells were serum starved for 20 h and stimulated with 50 ng/mL recombinant EGF for 10 min prior to harvest. For anisomycin studies, cells were serum-starved for 20 h and stimulated with 25 μg/mL anisomycin in dimethyl sulfoxide for 30 min prior to harvest. Cells were washed with PBS and incubated with TrypLE Express for 5 min at 37 °C. The resulting cell suspension was pelleted by centrifugation for 5 min at 200*g*. Pellets were washed with PBS and pelleted for 5 min at 200*g* for a total of two washes prior to lysis. Cells were lysed at 4 °C in Pierce IP Lysis Buffer containing Halt protease and phosphatase inhibitor cocktail. Lysates were clarified by centrifugation at 13,500 rpm for 15 min at 4 °C. Total protein content was determined by BCA. Aliquots were stored at −20 °C.

### Western Blotting

SDS loading dye (6×) containing dithiothreitol (DTT) was added to cellular samples and boiled at 95 °C for 5 min. Samples were loaded into precast 4–15% protein gels (mini-PROTEAN TGX, Bio-Rad) and electrophoresed for 30 min at 150 V. Proteins were transferred to a PVDF membrane using semidry transfer with an iBlot2 system (Invitrogen). Membranes were blocked for 1 h at room temperature in 5% bovine serum albumin in TBS-T (20 mM Tris, 150 mM NaCl, and 0.1% Tween), and primary antibody was added for overnight incubation at 4 °C with agitation. Blots were washed 3 times with TBS-T for 5 min at room temperature, and HRP-conjugated secondary antibody was added (1:2000 in BSA/TBS-T) for 50 min. After incubation with secondary antibody, blots were washed 3 times with TBS-T for 5 min at room temperature. Chemiluminescent signal was developed with Clarity Western ECL Substrate (Bio-Rad) and imaged using a Bio-Rad ChemiDoc XRS+. Antibody descriptions are found in Table S2. All blots were quantified using BioRad Image Lab version 6.0.1. Plots of signal intensity can be found in Figures S19–S22.

### *Shigella* Infection Assays

HeLa cells (ATCC) were maintained in high glucose DMEM (11965118, Thermo Fisher Scientific) supplemented with 10% heat-inactivated fetal bovine serum (FBS, Atlanta Biologicals), 100 IU/mL penicillin, and 100 μg/mL streptomycin (Life Technologies). One day prior to infection, HeLa cells were seeded at 8 × 10^5^ cells/well in 6-well tissue culture treated plates (Corning) with antibiotic-free DMEM supplemented with 10% FBS. Single red colonies of wild-type or Δ*ospF* *Shigella flexneri* 2457T expressing AfaI^26^ isolated from Congo red containing plates were inoculated into 2 mL of TCS (trypticase soy) broth and culture at 30 °C. The next day, each culture was diluted 1:50 into TCS broth and grown at 37 °C until an OD_600_ of 0.6 to 1.0. HeLa cells were then infected with *Shigella flexneri* resuspended in prewarmed low-glucose DMEM supplemented with 1% FBS at an MOI (multiplicity of infection) of 10. Plates were centrifuged at 2,000 rpm for 10 min to synchronize the infection and incubated at 37 °C for 0.5 h, after which each well was washed two times and then incubated in Hanks’ Buffered Salt Solution, 10% FBS, and 50 mM HEPES plus gentamicin (50 μg/mL). The plate was incubated for 0.5 h; then, the infected cells were treated with 0.5 mL of TrypLE. After incubation for 5 min, TrypLE was inactivated by adding the same volume of DMEM supplemented with FBS (1%) and gentamicin (50 μg/mL). After centrifugation at 2,000 rpm for 5 min, the cells were washed with 1 mL of PBS (phosphate-buffered saline) and then recentrifugated. The cells were lysed with Pierce IP Lysis buffer containing Halt protease and phosphatase inhibitor cocktail. All mammalian cell incubations were conducted in a 5% CO_2_ incubator at 37 °C, and all bacteria were grown in test tubes on a roller.

### Chemoproteomics Studies

Probe synthesis was performed as previously described.^23^ To 2 mg of HeLa lysate, TBS was added to bring lysate protein concentration to 1 μg/μL. Recombinant phospholyase was added to lysates and allowed 1 h to incubate at 26 °C to catalyze the formation of dehydrobutyrine in the proteome. After 1 h, probe was added to a final concentration of 1 mM and allowed to incubate for 18 h. Excess probe was removed using Pierce 3k MWCO Spin Concentrators. Briefly, 900 μL of TBS was added to probe-labeled lysates and then concentrated by centrifugation at 4000*g* for 1 h at room temperature. After 1 h, lysate was resuspended with 500 μL of TBS and concentrated by centrifugation at 4000*g* for 1 h at room temperature and repeated once more. The volume of probe-labeled lysate was then brought to 1 mL and added to 200 μL of Pierce Streptavidin magnetic beads; the sample was then incubated at 4 °C overnight with end-over-end mixing. Beads were washed three times with TBS-T and eluted by heating to 96 °C for 10 min in a solution of 3.3% sodium dodecyl sulfate (SDS), 200 mM DTT in Tris-HCl, pH 6.8. Eluted proteins were reduced with 5 mM DTT at 65 °C for 15 min with 1,000 rpm shaking and alkylated in the dark with 20 mM iodoacetamide at room temperature for 30 min. To remove SDS, samples were applied to S-trap microcolumns and prepared as described by the manufacturer. Proteins were digested on S-trap using 5 μg of sequencing-grade trypsin in 50 mM triethylammonium bromide buffer at 37 °C overnight. After eluting from S-trap columns, peptides were dried using a SpeedVac at 45 °C for 1 h and stored at −20 °C until use.

### Phospho-Protein Enrichment Studies

For phosphoproteomics studies, all cells were lysed with Takara ProteoGuard EDTA-Free Protease Inhibitor Cocktail and supplemented with 10 mM sodium fluoride. HeLa lysate (200 μg) was applied to commercial ion-metal affinity chromatography (IMAC) magnetic resin (TALON PMAC Magnetic Phospho Enrichment Kit) at 4 °C overnight and washed and eluted with buffers supplied by the manufacturer to capture the phosphoproteome. The eluted proteins were reduced as described above. Subsequently, the eluted phosphoproteins were applied to S-traps as described above and incubated with 5 μg of sequencing grade trypsin overnight at 37 °C. Peptides were dried using a SpeedVac at 45 °C for 1 h and stored at −20 °C until use.

### Whole Cell Lysate Proteomics Studies

HeLa lysate (250 μg) was denatured in 4 M urea in 50 mM triethylammonium bicarbonate (TEAB) and reduced and alkylated as described above. SDS was added to a final concentration of 3.3%, and samples were applied to S-trap midi columns and processed as described by the manufacturer. Proteins were digested on S-trap using 1:100 w/w sequencing-grade trypsin in 50 mM triethylammonium bromide buffer at 37 °C overnight. After eluting from S-trap columns, peptides were dried using a SpeedVac at 45 °C for 1 h and stored at −20 °C until use.

### Proteomic Study LC-MS Data Acquisition

Peptides were resuspended in 30 μL of 0.1% formic acid in water, and 2 μL of sample was injected onto a Vanquish Neo UHPLC system coupled with an Orbitrap Exploris 240 Mass Spectrometer for bottom-up proteomics with an EASY-Spray source interface between the LC and MS. Peptides were separated on EASY-Spray PepMap Neo columns (ES75750PN) with a maximum pressure set to 1500 bar. The system was controlled via Standard Instrument Integration for the Xcalibur software. All hardware and data acquisition software were purchased from Thermo Fisher Scientific. Mobile phase A was water with 0.1% formic acid (FA), and mobile phase B was 100% acetonitrile with 0.1% FA; all solvents were purchased from Thermo Fisher Scientific.

A 100 min linear gradient with a flow rate of 300 nL/min was used from 2%B to 20%B, followed by a 40 min linear gradient from 20%B to 35%B and finally a 10 min gradient from 35%B to 60%B. The autosampler was kept at a temperature of 4 °C, and column temperature maximum was set to 60 °C. The Orbitrap Exploris MS was operated in data-dependent acquisition (DDA) mode using a full scan with an *m*/*z* range of 350–1400 (resolution = 60,000, normalized AGC target = 300%, maximum injection time = 38 ms, dynamic exclusion for 100 s with a ± 10 ppm window). The intensity threshold for precursors was 8.0 × 10^3^. MS/MS spectra were acquired in DDA mode with a 3 s cycle time where each precursor above the intensity threshold was selected once before being added to the dynamic exclusion list for 100 s with a ±10 ppm window and subsequently fragmented for MS2 analysis via higher-energy collisional dissociation (HCD) using a normalized collision energy (NCE) of 28% (resolution = 11500, normalized AGC target = 50%, the maximum injection time = 35 ms).

### Proteomics Data Analysis

All proteomics data was acquired with three biological replicates (*n* = 3). Acquired Orbitrap Exploris 240 .raw DDA files were converted to .mzML, processed using FragPipe, and searched against a human Uniprot protein database containing common contaminants using MS Fragger.^27,28^ Database search criteria were as follows: fully tryptic with two missed cleavages, a precursor mass tolerance of 5 ppm, and a fragment ion tolerance of 5 ppm. Static modifications included carboxyamidomethylation of cysteines (+57.0214 Da). Dynamic modifications for chemoproteomics included phosphorylation on serine, threonine, and tyrosine (+79.966), chemical modification with TCEP-biotin on threonine (+589.246 Da), oxidation of methionine (+15.9949 Da), and protein N-terminal acetylation (+42.0106 Da). Peptide-spectrum matches were adjusted to a 1% false discovery rate (FDR) using Percolator.^29^ Label-free quantification was performed using IonQuant.^30^ All bioinformatic analysis of LC-MS/MS data was performed in the R statistical computing environment as previously described. Upregulation was defined as a protein that exhibited log_2_FC ≥ 1.0 and a *p*-value ≤ 0.05. Proportional Venn diagrams were created with https://www.deepvenn.com/.

### Proteomics Data Availability

The mass spectrometry proteomics data have been deposited to the ProteomeXchange Consortium via the PRIDE partner repository with the data set identifier PXD073985.

## Results and Discussion

### Assessing OspF Activity *in Vitro* on MAPK-Derived Peptides

OspF is known to target MAPKs ERK1/2 and p38, which share a strong sequence similarity with respect to activation loop phosphothreonine residues. Previous work in our lab alongside several other studies of OspF indicate that this enzyme may target additional phosphothreonine residues beyond the two reported on ERK1/2 and p38.^1,14,15^ Among the 14 MAPK family members,^31–33^ the activation loop exhibits a consensus sequence of ∼pT-E-Y-V∼ (Figure S1). This motif is shared by the MAPKs ERK5 and ERK7/8 but not by c-Jun N-terminal kinase (JNK). We therefore initiated this study by evaluating OspF-mediated phosphate β-elimination *in vitro* using synthetic phosphopeptides matching these activation loop sequences. We synthesized the peptides from ERK1/2 (peptide **1**, GFLpTEYV-NH_2_), ERK5 (peptide **2**, YFMpTEYV-NH_2_), ERK7/8 (peptide **3**, QAVpTEYV-NH_2_), and JNK (peptide **4**, FMMpTPYV-NH2). Using conditions that we previously optimized,^14^ we treated each peptide with OspF and evaluated an “apparent conversion” using LC-MS quantification of the resulting parent and Dhb-containing species (Figure 2A,B). We found that OspF was able to eliminate phosphate from 75.3 ± 1.3% of peptide **1** within 5 min, matching closely with our previous study.^14^ Peptides **2** and **3** were also eliminated by OspF (95.3 ± 0.36% and 69 ± 2.5%, respectively) (Figure 2C). However, incubation with OspF under identical conditions with peptide **4** only exhibited 4.3 ± 0.4% conversion to Dhb species after 5 min (Figure 2C). Compared to peptide **1**, peptide **4** exhibits much slower elimination (Figure 2D) but eventually reaches near quantitative conversion to Dhb after 24 h (Figure S2). We extended this peptide model to interrogate whether SpvC, a related phospholyase from *Salmonella enterica* Typhimurium, would exhibit similar behavior. SpvC is known to share a high degree of homology with OspF and uses the same elimination mechanism on many of the same substrates, such as ERK1/2 and p38 (Figure S3).^8–10,13,34^ Using SpvC, we observed double the extent of elimination of peptide **4** after 5 min (11.9 ± 1.1%) compared to that observed with OspF (Figure 2D). Despite the modest levels of conversion at early time points, SpvC was eventually able to convert pJNK to its corresponding Dhb species after 24 h (Figure S2). Together, these results suggest that JNK is a poorer phospholyase substrate than other MAPKs.

**Figure 2:**
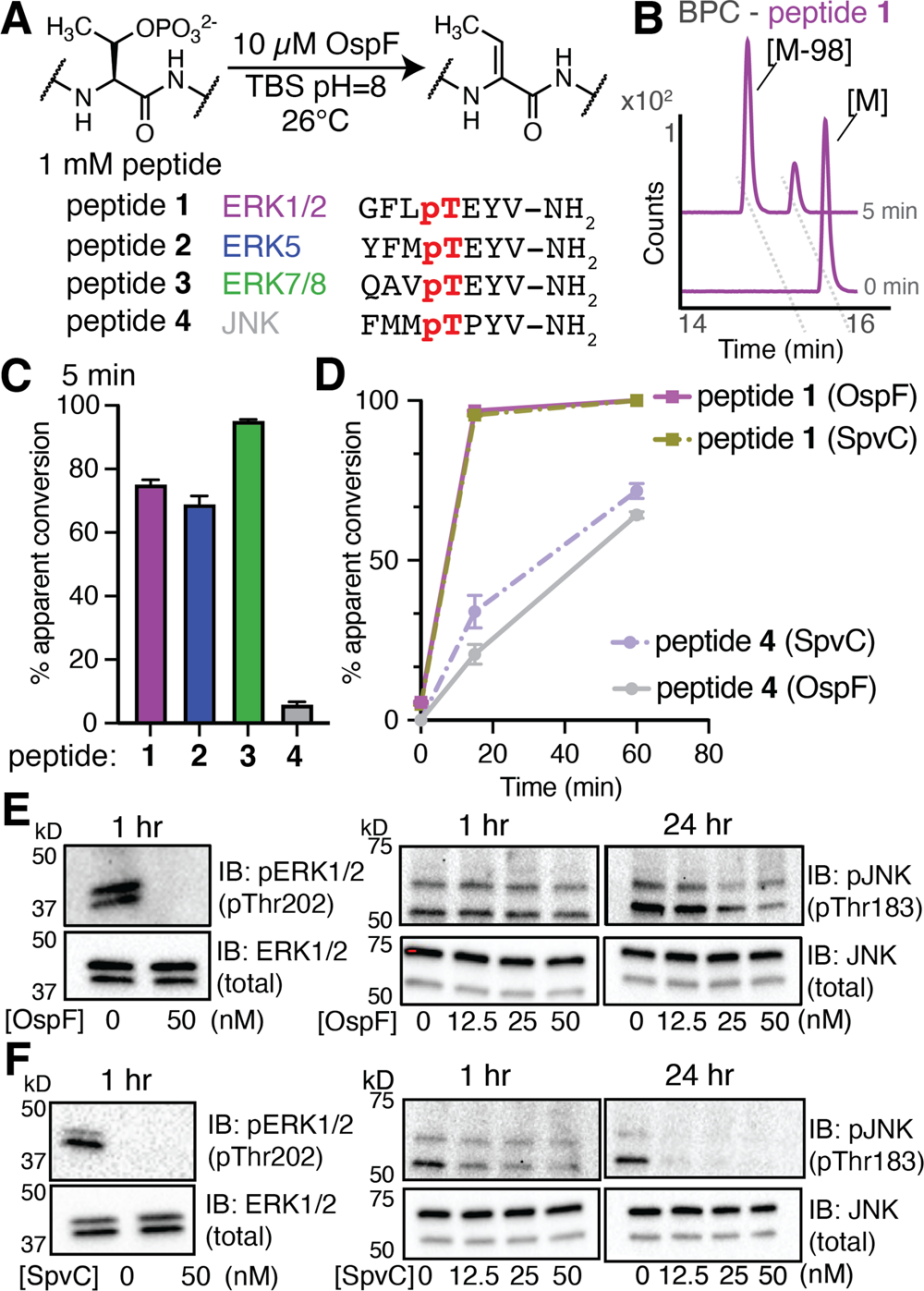
OspF eliminates phosphate from MAPK-derived peptides. (A) To assess phosphate β-elimination, we synthesized several phosphopeptides corresponding to MAPK activation loop sequences treated with 10 μM recombinant OspF and monitored via LC-MS. (B) A representative base peak chromatogram (BPC) depicts the emergence of a new peak after the treatment with OspF, corresponding to the Dhb species associated with peptide **1**. (C) Apparent conversion for peptides **1** through **4** after 5 min of incubation with 10 μM OspF, plotted as mean (*n* = 3) ± standard error (SE). (D) Time-course measurements of peptide **1** and peptide **4** after incubation with either OspF or SpvC, plotted as mean (*n* = 3) ± SE. Western blotting using total and phospho-specific antibodies reveals the ability of either OspF (E) or SpvC (F) to eliminate ERK1/2 (left, *n* = 4) and JNK (right, *n* = 3) in EGF-stimulated HeLa lysates.

However, since the phosphopeptides used in our *in vitro* assay lack the three-dimensional structure and targeting domains that are known to help dock phospholyases to their substrates, we sought to confirm that these findings extended to full-length proteins. Thus, we exposed serum-starved, EGF-stimulated HeLa cell lysates to recombinant OspF and SpvC and evaluated the phosphorylation of ERK1/2 or JNK using phospho-specific antibodies. Expectedly, we saw complete removal of the phospho-ERK1/2 signal within 1 h for both phospholyases (Figure 2E,F left). We used the same approach to evaluate the phospho-JNK signal, and hardly any decrease in phospho-JNK signal was observed after 1 h of treatment with OspF, while SpvC led to a modest loss of pJNK signal (Figure 2F, right). After 24 h, SpvC nearly completely erased the pJNK signal at all concentrations tested. In contrast, OspF only led to modest elimination of pJNK and only at higher concentrations of enzyme (Figure 2E, right). Additionally, stimulating HeLa cells with anisomycin, a potent activator of JNK phosphorylation, revealed that both SpvC and OspF were clearly able to directly modify pJNK after 1 h with 50 nM SpvC completely erasing phospho-JNK, whereas 50 nM OspF merely reduced it (Figure S4). This shows that JNK is a relatively poor phospholyase substrate, especially for OspF. Some prior work has suggested that pJNK is an OspF substrate, but other reports suggest that OspF silences JNK activity by eliminating phosphate from upstream kinases instead.^34–36^ Our results show that, while OspF could modify JNK, it does so rather inefficiently. This lends support to the idea that OspF’s impact on JNK phosphorylation is likely to be indirect during infection. Additionally, our results revealed that OspF led to unexpected modification of an atypical MAPK substrate (peptide **3**) that rivaled that of classical MAPKs like ERK1/2 and p38. This observation raised broader questions about the extent to which phospholyase activity extends beyond its previously reported substrates as many classes of phosphoproteins are involved in immune functions that are activated during *Shigella* infection. Thus, we sought to explore OspF’s activity within the mammalian proteome to identify other substrates.

### Developing a Chemoproteomic Workflow to Assess OspF Activity

To date, only one study has been published that examines changes in the phosphoproteome in response to OspF.^15^ While this study revealed widespread OspF-dependent changes in the phosphoproteome, it could not differentiate between those that were due to the direct effects of OspF exposure (phosphate β-elimination) or indirect inactivation of downstream phospho-signaling pathways. We sought to develop an enrichment-based chemoproteomics method using a chemical probe (TCEP-biotin, probe **1**) that we previously reported, enabling enrichment of OspF-modified proteins and the identification Dhb-labeled sites (Figure 3A,B,C).^23^ To identify Dhb-modified proteins, we spiked serum-starved, EGF-stimulated HeLa cell lysate with or without wild-type OspF (OspF^WT^) followed by treatment with probe **1** and subsequent pull-down with streptavidin. We then used label-free quantitation (LFQ) to identify proteins enriched only upon OspF treatment by monitoring upregulated proteins (log_2_FC > 1, *p*-value < 0.05) (Figure 3D). We titrated OspF from 50 to 0.5 nM and monitored proteins upregulated relative to untreated probe **1**-labeled lysates. Inspection of protein expression levels (Figure S5) revealed that the most abundant MAPK in our HeLa lysates was ERK2, so we sought to monitor this enzyme and not p38 or other MAPKs since they were sparingly abundant, and thus, we would not expect to see them pulled down. As expected, ERK2 had a large log_2_FC across all conditions (1.40, 2.47, and 1.61, respectively), though it fell outside the threshold for statistical significance at the highest enzyme concentration (Figure S6). Because ERK2 was upregulated at two of three OspF concentrations, we decided to query IDs that were likewise upregulated for at least two concentrations, producing a list of 385 protein IDs of putative Dhb-modified proteins (Figure 3E).

**Figure 3:**
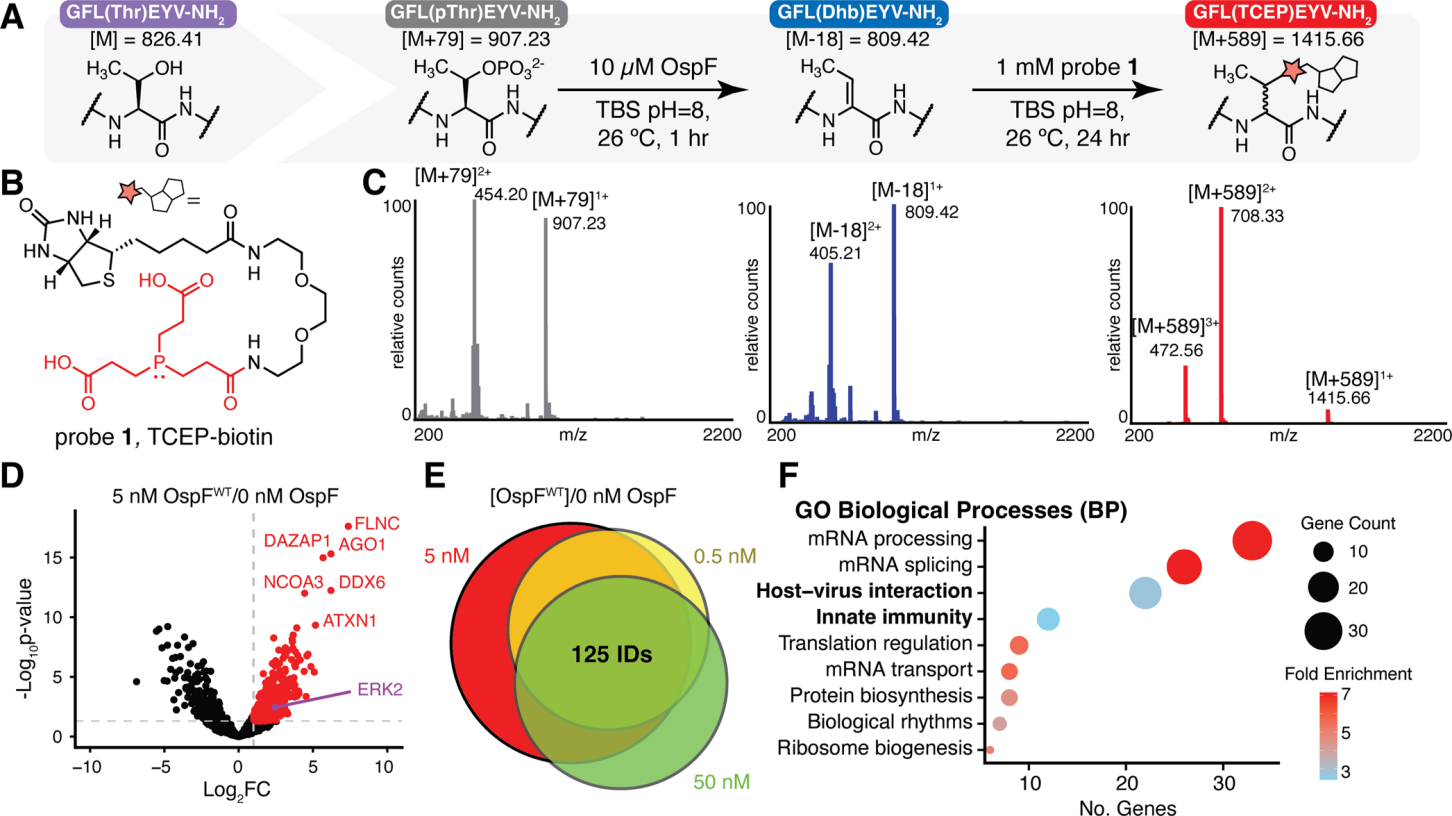
Profiling recombinant OspF’s activity in the proteome using an activity-based probe. (A) A recently reported phosphine probe, structure shown in (B), is able to form covalent adducts with Dhb derived from peptide **1**, thus allowing us to chemically label Dhb formation. (C) Representative spectra depicting peptide **1**’s β-elimination and subsequent β-phosphonium adduct with probe **1**. (D) After treating HeLa lysate with OspF and probe **1**, label-free quantitation (LFQ) analysis relative to untreated lysates reveal a set of proteins that exhibit upregulation (log_2_FC > 1.0 and *p*-value < 0.05 (empirical Bayes-moderated *t* test)) after OspF treatment. (E) Titration of OspF concentrations reveal a subset of proteins that exhibited upregulation at all concentrations of OspF. (F) Gene ontology (GO) biological process (BP) analysis reveals terms associated with proteins upregulated at 2 of 3 OspF concentrations (FDR < 0.1).

Our previous work showed that addition of the TCEP-biotin probe leads to nonspecific background signal in cell lysates, likely due to a combination of its potent nucleophilicity and redox activity.^23^ To control for any potential off-target effects, we compared treatments with wild-type OspF to those with a catalytically inactive variant (OspF^K134A^). This approach provided greater stringency, allowing us to better understand the hits that were dependent on OspF’s catalytic activity. Encouragingly, in this comparison, ERK2 fell well below the threshold for enrichment (log_2_FC = 0.62, *p*-value = 0.08), indicating that our chemoproteomic strategy reports on phospholyase activity (Figure S6C). Using this approach, we narrowed our list to 194 hits. We likewise queried this list of 194 hits against proteins nonspecifically enriched in the presence of streptavidin affinity support as catalogued in the leading contaminant repository database (Figure S7).^37^ We filtered our list of hits against a stringent list of nonspecific streptavidin binders that were found in at least eight independent streptavidin pulldown experiments. We were encouraged to find that 63.4% of the hits we identified passed this filtering step. However, as the known OspF substrate ERK2 is flagged as a nonspecific streptavidin binder using these criteria, we opted not to remove any hits based on this analysis to avoid removal of genuine substrates. With a high confidence list of Dhb candidates in hand, we performed gene ontology biological process (GO BP) analysis to define OspF’s proteomic signature in EGF-stimulated HeLa lysates by selecting terms that had FDR < 0.1. This analysis revealed expected terms related to immunity (innate immunity, host–virus interaction) but likewise revealed terms related to RNA splicing (mRNA splicing, mRNA processing) and protein biosynthesis (Figure 3F). These GO results suggest that OspF may perturb signaling pathways beyond MAPKs in the host proteome, though these results may be biased by the choice of EGF as a phospho-signaling stimulus, the high concentrations of enzyme used, and the absence of full type III secretion system (T3SS) machinery.

### Investigating the Role of OspF’s D-Domain on Catalysis and Specificity

Like other phospholyases, OspF has a canonical N-terminal D-motif containing basic and hydrophobic residues that are essential for MAPK-binding.^9,34,38–40^ Previous work has shown that D-domain mutations disrupt docking to MAPKs, yet it is unclear if this domain confers any consequence for OspF’s biochemical activity. This prompted us to investigate the extent to which the OspF D-domain influences substrate recognition and/or catalysis. First, to determine the impact of the D-domain on OspF’s biochemical activity, we expressed and purified a variant of OspF with a previously reported N-terminal deletion (ΔN26) that removes its entire D-domain (Figure 4A).^34^ We found that the OspF^ΔN26^ variant exhibited diminished activity *in vitro* against peptide **1** (44.1 ± 3.8% apparent conversion) compared to that of OspF^WT^ (75.3 ± 1.3%) (Figure S8). Similarly, OspF^ΔN26^ could not eliminate phospho-ERK1/2 in serum-starved, EGF-stimulated HeLa cell lysates after 1 h, whereas OspF^WT^ was able to promote elimination even at low concentrations (Figure 4B).

**Figure 4:**
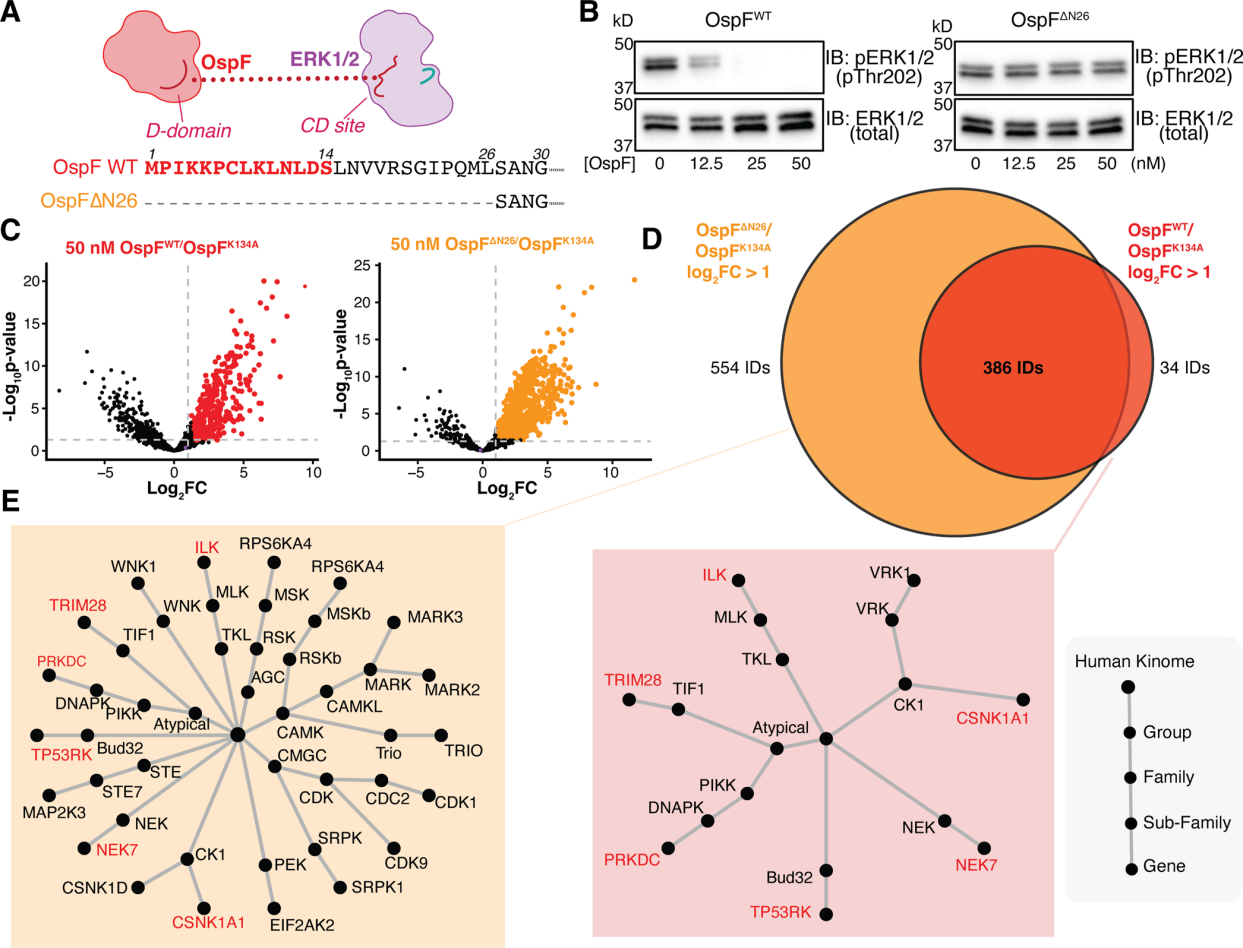
OspF’s activity and specificity depend on its D-domain. (A) Sequence alignment of OspF^WT^ and OspF^ΔN26^. (B) HeLa cells were serum-starved and stimulated with 50 ng/mL EGF for 10 min. After harvesting and lysing, lysates were spiked with 0–50 nM of either OspF^WT^ or OspF^ΔN26^ and incubated for 1 h at 26 °C. Lysates were probed for pERK1/2 and total ERK1/2. (C) LFQ analysis of HeLa lysates spiked with either OspF^WT^ or OspF^ΔN26^ reveal proteins enriched relative to catalytically dead OspF^K134A^ with the upregulation threshold corresponding to log_2_FC > 1.0 and *p*-value < 0.05 (empirical Bayes-moderated *t* test). (D) Upregulated proteins found in the enzymes of both OspF^WT^ and OspF^ΔN26^ depicted as a Venn diagram. (E) Radial dendrograms depict kinase phylogenies for the enzymes of both OspF^WT^ and OspF^ΔN26^ with kinases shared between both enzyme variants highlighted in red.

Because we observed a decrease in activity for OspF^ΔN26^ against ERK1/2, we sought to further characterize how D-domain deletion would impact OspF’s proteomic footprint. Using our LFQ workflow, we treated serum-starved, EGF-stimulated HeLa lysates with either OspF^WT^ or OspF^ΔN26^ and monitored the log_2_FC of ERK2 after enrichment relative to untreated lysates. We found that ERK2 was not a hit for this enzyme variant (log_2_FC = 1.07, *p*-value = 0.24, not significant), consistent with our observation of OspF^ΔN26^’s diminished activity against peptide **1**. However, we found that OspF^ΔN26^ appears to interact with many additional proteins compared to OspF^WT^ based on the number of hits above the log_2_FC threshold relative to OspF^K134A^. Specifically, although 91.7% of upregulated proteins (log_2_FC > 1 and *p*-value < 0.05) after treatment with OspF^WT^ were also found upregulated for OspF^ΔN26^, an additional 554 proteins were found to be upregulated only after treatment with OspF^ΔN26^ (Figure 4C,D). This behavior was unexpected due to the attenuated biochemical activity that we observed with peptide **1** and OspF^ΔN26^. To gauge whether these trends tracked with enhanced biochemical activity or a broader substrate scope for OspF^ΔN26^, we applied a kernel density estimation (KDE) to protein abundances that were dependent on OspF treatment using the log_2_ abundance ratio of OspF^WT^/OspF^ΔN26^. The rightward skew of our KDE plot, including several peaks greater than 0, builds support for the idea that OspF^ΔN26^ likely accesses more of the proteome but does so less productively (Figure S9).

To further understand this behavior, we focused on the kinase hits for each variant. We produced a kinase phylogenetic tree to visualize which kinase groups, families, and subfamilies were captured by both enzymes’ activity.^41^ We observed that OspF^WT^ engaged seven kinases, six of which were also hits for OspF^ΔN26^. In contrast, OspF^ΔN26^ engaged a wider range of kinases than OspF^WT^ (Figure 4E), suggesting that removal of the D-domain expands substrate accessibility despite decreasing its biochemical activity toward ERK1/2. To verify that the increased positive signal for Dhb in our chemoproteomics with OspF^ΔN26^ was not reflective of this mutant’s biochemical activity and more likely explained by substrate accessibility, we synthesized a peptide sequence pertaining to one of the top hits by log_2_FC from both OspF^WT^ and OspF^ΔN26^, and we found the same activity trends as observed with peptide **1** with OspF^WT^ more effectively (Figure S10).

### Chemoproteomic Profiling of OspF Targets in *Shigella* Infection

During an infection, OspF works in concert with nearly 30 other *Shigella* type III secreted effectors, potentially limiting the biological relevance of our recombinant enzyme studies.^42,43^ Therefore, we next used our chemoproteomic platform to identify OspF substrates in *Shigella*-infected cells using a platform that delivers OspF into HeLa cells at physiologically relevant levels.^44^ Specifically, we infected HeLa cells with wild-type (WT) or Δ*ospF Shigella*, a strain that no longer encodes OspF. After labeling lysates with probe **1**, we confirmed that ERK1/2 was specifically modified and biotinylated in lysates of WT *Shigella* infected cells (Figure 5A,B). We proceeded to perform proteomic analyses on probe-labeled lysates from cells infected with each strain. We then compared the log_2_FC of proteins immunoprecipitated from WT relative to Δ*ospF* lysates. ERK2 was upregulated at a log_2_FC of 2.65 (*p*-value = 5.92 × 10^–7^). Strikingly, ∼70% of our hits from our recombinant enzyme studies were also upregulated (log_2_FC > 1, *p*-value < 0.05). However, many new proteins exhibited a high log_2_FC under infectious conditions, so we chose to use a more stringent cutoff of log_2_FC > 2.5 to focus on proteins for follow-up studies, which limited the hit list to 392 proteins, 11 of which were also marked as hits in our recombinant enzyme studies (including ERK2) (Figure S11).

**Figure 5:**
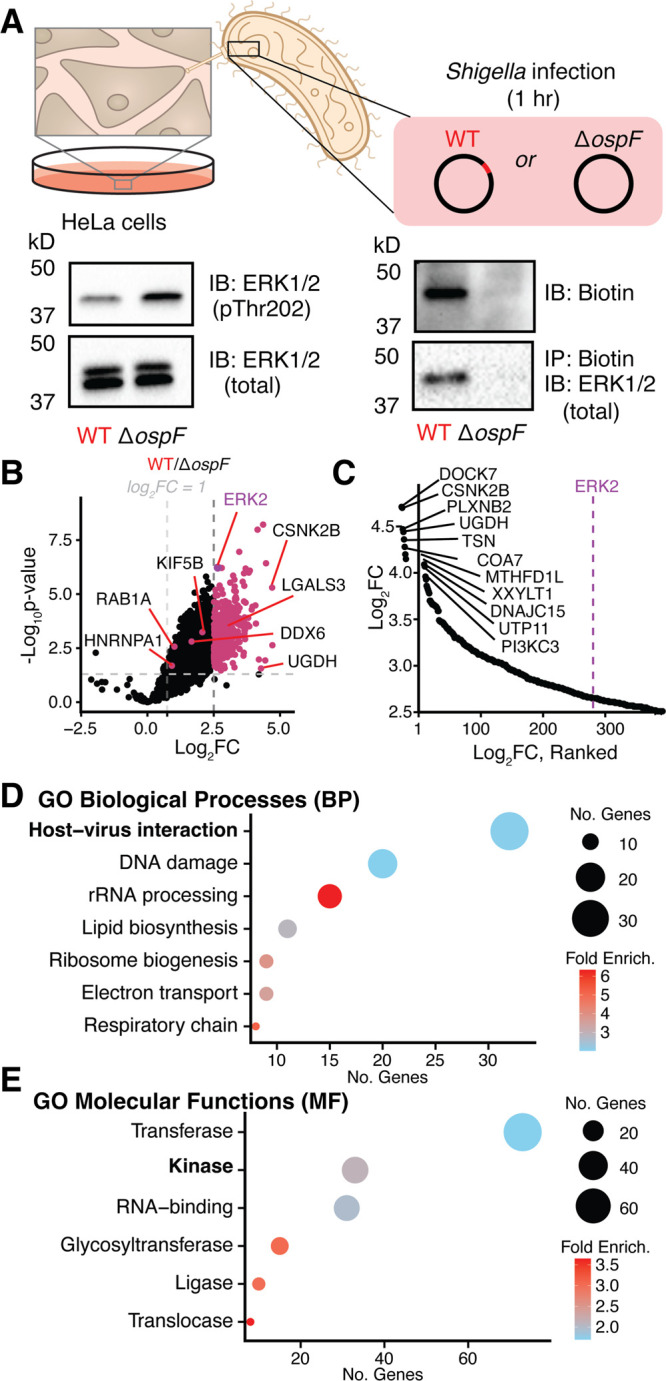
Profiling OspF’s effect on the infected proteome. (A) To profile OspF’s activity during *Shigella* infection, we took cells infected with either wild-type (WT) or OspF knockout (Δ*ospF*) *Shigella flexneri*, shown as a schematic. Using lysed, infected cells, Western blotting revealed an OspF-dependent loss pERK1/2 signal alongside no change in total ERK1/2 (*n* = 3). Incubation of these lysates with probe **1** reveals a band at ∼42 kDa after probing with biotin antibody (*n* = 3), which is then revealed via immunoblot (*n* = 2) to be ERK1/2 after immunoprecipitation with streptavidin beads. (B) Volcano plot displaying proteins enriched after WT infection relative to Δ*ospF* infection. Two log_2_FC thresholds for upregulation were considered (log_2_FC > 1.0, *p*-value < 0.05 and log_2_FC > 2.5, *p*-value < 0.05, empirical Bayes-moderated *t* test). (C) Log_2_FC rank analysis reveals the top upregulated proteins after infection with *Shigella* expressing OspF. (D) GO biological processes and (E) GO molecular function analyses reveal terms associated with OspF-dependent upregulation during infection (FDR < 0.1).

Surprisingly, we found that ERK2 did not exhibit the highest log_2_FC. Over 250 of the 392 proteins exhibited a higher log_2_FC (Figure 5C). Interestingly, biological process GO enrichment analyses (FDR < 0.1) revealed that the host–virus interaction had the largest number of hit proteins (Figure 5D), while molecular function (GO MF) analysis suggested that transferases and kinases are the most targeted by OspF during an infection (Figure 5E). In parallel, we performed functional enrichment clustering analysis using DAVID to reveal distinct clusters of terms across multiple gene ontology databases. This approach revealed 30 statistically significant clusters (Table S4, Figure S12), including nuclear localization, cytoplasmic localization, and nucleotide-binding/kinase activity, as the clusters with the highest enrichment scores. We likewise found clusters related to broader processes associated with *Shigella* infection such as membrane ER-Golgi vesicle mediated transport as well as ubiquitin and ubiquitin-like ligase conjugation. These align with other known *Shigella* virulence factor functions, as the effector IpaH is an E3 ligase,^45^ OspG is a kinase that disrupts host ubiquitination machinery through phosphorylation,^46^ and OspF itself is SUMOylated by host machinery to translocate to the nucleus during infection.^47^

Using our results from this clustering analysis, as well as monitoring the overall trends shared between both our *in vitro* recombinant enzyme and infection studies, we chose to follow up on a subset of hits that encompassed diverse functional processes related to *Shigella* infection and OspF activity (Figure S13). First, we chose CSNK2B (casein kinase II subunit β), a kinase outside of the MAPK family that reflected a broad array of functional processes observed in our data set. Next, we selected DDX6 (RNA-dependent DEAD box helicase 6), a helicase linked to the formation of RNA stress granule response, which is targeted during infection with *Shigella*.^48,49^ Further, we selected RAB1A (Ras-associated binding protein 1A), which interacts with VirA, another *Shigella* type III secreted effector.^50^ Likewise, we selected UGDH (UDP-glucose dehydrogenase), which exhibited a high log_2_FC and was localized with a high resolution probe **1** dynamic modification (Figure S14). We additionally included other hits that exhibited high log_2_FC from exposure to recombinant or *Shigella*-secreted OspF, including kinesin family member 5B (KIF5B), galectin-3 (LGALS3), and heterogeneous nuclear ribonucleoprotein A1 (HNRNPA1) that were also reflective of various functional clusters revealed from our analysis of hits.

As a first test, we prepared a small group of phosphopeptides based on these hits (peptides **6**–**14**, Figure 6A), which were incubated with recombinant phospholyases *in vitro*, akin to our study of MAPK-derived peptides (Figure 2). Since OspF has been shown to modify pSer *in vitro*,^14^ we sought to include several pSer-containing sequences in this group of synthetic peptides. Using this approach, we found that peptides derived from CSNK2B, RAB1A, and DDX6 exhibited modest reactivity (>65% apparent conversion to Dhb species) when treated with OspF (Figure 6B). These three top-performing hits, respectively, correspond to high-ranking log_2_FC during infection (CSNK2B) and after exposure to recombinant enzyme (DDX6). Curiously, RAB1A exhibited only moderate upregulation (log_2_FC = 1.20, *p*-value = 3.08 × 10^–3^) yet represented many different functional clusters (Figure S10), exhibited one of the strongest decreases in phospho-signal after exposure to recombinant OspF (Figure S15), and was localized with a Dhb modification at the site corresponding to peptide **6** (Thr137) in unenriched *Shigella*-infected lysates in the absence of probe **1** (Figure S16). Further, we found that OspF exhibited sequence selectivity toward a peptide derived from pThr37 of CSNK2B (peptide **7**), whereas our sequence derived from pSer209 (peptide **8**)–CSNK2B’s most characterized phosphosite^51^–proceeded with minimal conversion (<2%) to Dhb species.

**Figure 6:**
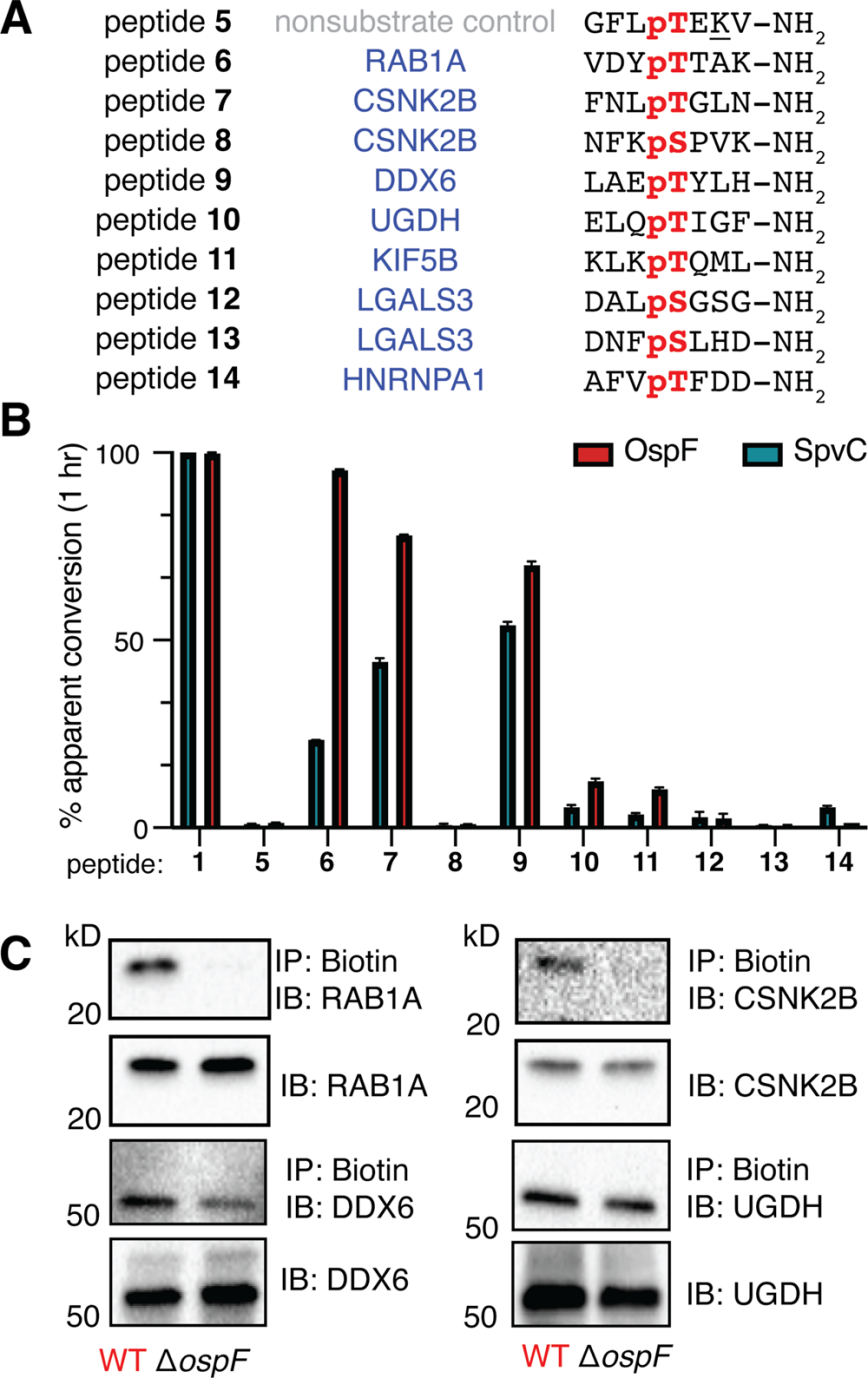
Validation of the modification of OspF beyond MAPKs using phosphopeptides. (A) Sequences of peptides synthesized based on chemoproteomic hits. (B) Incubation of peptides with 10 μM recombinant OspF or SpvC reveal apparent conversion to the corresponding Dhb species after 1 h, plotted as mean (*n* = 3) ± SE. (C) Western blot (*n* = 2) after incubating infected lysate with probe **1** and streptavidin beads reveals differential pulldown of proteins identified via chemoproteomics.

Encouragingly, nearly all peptides exhibited greater conversion to Dhb with OspF than SpvC, the related *Salmonella* homologue. As our results with peptide **4** and JNK revealed the opposite trend, this finding suggests that our workflow reports on authentic OspF activity. For those proteins for which antibodies are commercially available (RAB1A, CSNK2B, DDX6, and UGDH), we next tested whether biotinylated variants were immunoprecipitated using streptavidin beads from lysates of cells infected with WT or Δ*ospF Shigella*. We observed that RAB1A and CSNK2B were exclusively immunoprecipitated from WT *Shigella*-derived lysates, while enhanced levels of DDX6 were precipitated from WT vs Δ*ospF Shigella*-derived lysates. Consistent with the phosphopeptide screen, in which UGDH led to far lower levels of apparent conversion, UGDH was precipitated at equivalent levels from lysates, suggesting that its biotinylation may have been an off-target effect of the chemical probe.

As an ideal next step, we would test whether OspF targets the removal of these phosphosites by using phospho-specific antibodies (Figure 6C). However, such antibodies are not available for DDX6 or RAB1A, as the phosphorylation of both these proteins are not widely appreciated. The few RAB1A phosphorylation studies conducted have focused on Thr75.^52^ Rather, we have observed Dhb formation at Thr137, a modification that has been previously reported but never characterized.^53^ Similarly, phosphorylation of DDX6 has not been reported in any functional context, despite many sites experimentally observed in large scale phosphoproteomics studies, including the site corresponding to the sequence of peptide **8** (Thr417).^53^ CSNK2B phosphorylation has largely been studied within the context of Ser2 and Ser209, which serve regulatory functions.^51,54^ Our results, however, point to Thr37 as an OspF-sensitive phosphosite. Thus, not surprisingly, we did not observe any changes in the phosphorylation state of pSer209 upon recombinant or *Shigella*-derived OspF exposure (Figure S17), using the solely available phospho-specific CSNK2B. Nevertheless, prior large-scale studies of the phosphoproteome^55^ also previously detected phosphorylation of Thr37. To further confirm the accessibility of Thr37 as a regulatory site during *Shigella* infection, we used AlphaFold3 to predict interactions of the CSNK2 tetrameric complex with OspF.^56^ Encouragingly, in the AlphaFold3 model, the active site of OspF was closest to pThr37. Specifically, the Nε of OspF’s catalytic Lys was predicted to be 17.25 Å from Cα of pThr37, whereas pSer2 and pSer209 were both predicted to be over two times farther away (49.76 and 39.03 Å, respectively) (Figure S18). These results further support our findings that pThr37 is eliminated by OspF, revealing a potential immune function for a phosphosite that currently has not been characterized on CSNK2B. As the CSNK2 complex is implicated in various bacterial infections,^57–59^ we anticipate that this finding lays the groundwork for the biological study of CSNK2B’s role in *Shigella* infection, which has not yet been recognized.

## Conclusion

This study describes the first chemoproteomic profile for phospholyase-mediated Dhb formation, using recombinant OspF both *in vitro* and in *Shigella* infection. Together with the use of synthetic phosphopeptides to define the *in vitro* biochemical activity of the phospholyase OspF, these studies reveal a wide range of OspF activity, both within the MAPK family and beyond, which has not been previously appreciated. Specifically, we show that OspF exhibits selectivity within the MAPK family, leading to substantial Dhb formation on ERK1/2, ERK7/8, and ERK5 but not JNK. Using a recently reported chemical probe, we discovered that OspF modifies many proteins that are outside of the MAPK family, including CSNK2B, RAB1A, and DDX6. To date, none of these proteins have been identified as putative substrates for OspF. As these proteins are heavily involved in pathways related to cytokine and RNA stress and host autophagy, we suspect that they may be important in *Shigella* infection, allowing for bacteria to exploit host machinery and propagate in host cells once eliminated.

However, we note that biological validation of the targets of OspF that does not rely on chemical probes is particularly challenging. Not only is there limited commercial availability of specific pSer/pThr antibodies, despite making up the majority of the phosphoproteome relative to pTyr,^60^ but also the mass changes for loss of water and OspF phosphate β-elimination are identical, making it difficult to discern the difference via unbiased proteomic analysis.^16–22^ Our previously developed chemical probe allows us to circumvent such issues by selectively labeling Dhb modifications and identifying OspF hits. However, this probe still has some drawbacks, especially the potential for nonspecific background, which could be addressed through further chemical tuning of a phosphine probe or the identification of alternative labeling chemistry in future work. Along with the development of new strategies for hit validation, we expect that this approach could provide important insights into the pathways involved in previously unrecognized host–pathogen interactions during *Shigella* infection. Such insights may offer access to new strategies for combating the looming threat posed by antibiotic resistance.

Outside of *Shigella* infection, OspF and its phospholyase family members are attractive targets as potential molecular tools that catalyze an “orthogonal” transformation, which is not currently thought to occur in the healthy human proteome.^14^ The orthogonality of this transformation represents an untapped scaffold for chemical tools that exploit Dhb’s electrophilicity to study phosphorylation signaling and host–pathogen interactions. Here, we show that phosphine-based chemical probes are valuable for tracking Dhb formation in a cellular context. Our study also shows the substantial role that OspF’s D-domain plays, not only as a substrate docking element but also in modulating its biochemical activity. Our future work will continue to explore the biochemical and structural requirements for OspF’s activity and proteome accessibility of OspF, which can serve as a framework to harness its activity for precise inhibition of phosphorylation pathways and/or the site-specific installation of electrophilic chemical handles that can be further modified. Altogether, our findings lay the groundwork for the design of novel phospholyase-based tools for a range of chemical and synthetic biology applications.

## Supplementary Material







## Notes

Additional experimental details, materials, methods; MAPK activation loops; overnight incubation of pJNK peptide with phospholyases; OspF and SpvC sequence alignment; Western blots of pJNK/total JNK; protein abundance for HeLa lysate after EGF stimulation and bacterial infection; volcano plots compared to 0 nM OspF; Venn diagram for nonspecific binders; elimination of peptide **1** between OspF^WT^ and OspF^ΔN26^; kernel density estimate between OspF^WT^ and OspF^ΔN26^; elimination of peptides **1** and **8** between OspF^WT^ and OspF^ΔN26^; upregulated proteins after recombinant and *Shigella*-delivered OspF exposure; functional enrichment clusters; score distribution for enrichment clusters; functional enrichment clustering contexts for genes followed up during peptide studies; representative EIC and MS2 spectrum for UGDH^TCEP^; volcano plot for 50 nM OspF and IMAC; representative EIC and MS^2^ spectrum for RAB1A^Dhb^; Western blots of pCSNK2B/total CSNK2B; AlphaFold images of OspF with CSNK2A/B; densitometry for western blots from JNK, D-domain, infection chemoproteomics, and hit validation studies (PDF)
Summary of proteomics data set (XLSX)
Full DAVID clustering analysis results (XLSX)

## Notes

Proteins identified in the text of this manuscript are primarily referred to by their gene name. UniProt identifiers for proteins referenced in the main text of this study are as follows: RAB1A (P62820), DDX6 (P26196), LGALS3 (P17931), CSNK2B (P67870), KIF5B (P33176), HNRNPA1 (P09651), UGDH (O60701), MAPK1 (P28482), MAPK14 (Q16539), MAPK8 (P45983), MAPK7 (Q13164), MAPK15 (Q8TD08), OSPF (Q8VSP9), and SPVC (P0A2M9). Additional UniProt accession information for MAPKs can be found in Figure S1. For additional proteomics hits UniProt accession information, identifiers can be found in Table S3.
