# Supplementary material for: Profiling Selectivity for the Shigella Virulence Factor OspF
Source: Biochemistry. 2026 Apr 22;65(9):1495–506. doi: 10.1021/acs.biochem.6c00109 (PMC13151050; doi:10.1021/acs.biochem.6c00109)
Supplement: Supplementary file 1 [file bi6c00109_si_001.pdf]

# Supporting Information

# Profiling Selectivity for the *Shigella* Virulence Factor OspF

Nicholas P. McCurtin‡[1], Ariana Gazaferi‡[1], Noah D. Novick[1], Kyungsub Kim[2], Cammie F. Lesser[2,3], and Rebecca A. Scheck*[1]

[1]Department of Chemistry, School of Arts and Sciences, Tufts University, 62 Talbot Avenue, Medford, Massachusetts 02155, USA
[2]Department of Molecular Biology and Microbiology, Tufts University School of Medicine, 150 Harrison Avenue, Boston, Massachusetts 02111, USA
[3]Levy Center for Integrated Management of Antimicrobial Resistance, South Cove 502, 136 Harrison Ave, Boston, MA. 02111, USA
‡Equal contribution. Contact: rebecca.scheck@tufts.edu

**Materials and Methods** S2
**Supporting Figures and Tables**
**Table S1.** Peptide masses S5
**Table S2.** Antibodies S6
**Table S3.** UniProt accession information S7
**Figure S1.** MAPK activation loops S8
**Figure S2.** Overnight incubation of pJNK peptide with phospholyases S9
**Figure S3.** OspF and SpvC sequence alignment S10
**Figure S4.** Western blots of pJNK/total JNK S11
**Figure S5.** Protein abundance for HeLa lysate after EGF stimulation and bacterial infection S12
**Figure S6.** Volcano plots compared to 0 nM OspF S13
**Figure S7.** Venn diagram for nonspecific binders S14
**Figure S8.** Elimination of peptide 1 between $OspF^{WT}$ and $OspF^{\Delta N26}$ S15
**Figure S9.** Kernel density estimate (KDE) between $OspF^{WT}$ and $OspF^{\Delta N26}$ S16
**Figure S10.** Elimination of peptide 1 and 8 between $OspF^{WT}$ and $OspF^{\Delta N26}$ S17
**Figure S11.** Upregulated proteins after recombinant and *Shigella*-delivered OspF exposure S18
**Table S4.** Functional enrichment clusters S19
**Figure S12.** Score distribution for enrichment clusters S20
**Figure S13.** Functional enrichment clustering contexts for genes followed up during peptide studies S21
**Figure S14.** Representative EIC and $MS^2$ spectrum for $UGDH^{TCEP}$ S22
**Figure S15.** Volcano plot for 50 nM OspF and IMAC S23
**Figure S16.** Representative EIC and $MS^2$ spectrum for $RAB1A^{Dhb}$ S24
**Figure S17.** Western blots of pCSNK2B/total CSNK2B S25
**Figure S18.** AlphaFold images of OspF with CSNK2A/B S26
**Figure S19.** Densitometry for western blots from JNK study S27
**Figure S20.** Densitometry for western blots from D-domain study S28
**Figure S21.** Densitometry for western blots from infection chemoproteomics study S29
**Figure S22.** Densitometry for western blots from hit validation study S30

**References** S31

**General.** All chemical reagents were of analytical grade, obtained from commercial suppliers and used without further purification unless otherwise noted. Water used in biological procedures was distilled and deionized using an Arium® pro purification system (Sartorius). All statistical analysis was conducted using Prism GraphPad.

**Peptide Synthesis.** Amidated peptides were synthesized on Rink Amide MHBA resin on a 0.05 mmol scale following standard Fmoc peptide synthesis procedures in fritted syringes. Fmoc was deprotected using 20% piperidine in dimethylformamide (DMF) For more sensitive peptides, a 2% solution of 1,8-diazabicyclo[5.4.0]undec-7-ene (DBU) in DMF was used to deprotect Fmoc to avoid base-catalyzed elimination. Fmoc-protection was accomplished by two iterative washes with one full syringe volume of DBU for 5 minutes at room temperature, followed by four iterative washes with one full syringe volume of DMF. For coupling, 5 eq. amino acid was added to 5 eq. O-(benzotriazol-1-yl)-N,N,N′,N′-tetramethyluronium hexafluorophosphate (HBTU), 5 eq. hydroxybenzotriazole (HOBt), and 10 eq. N,N-diisopropylethyllamine (DIPEA) in DMF and allowed to pre-activate for 5 minutes. The coupling solution was added to the fritted syringe and allowed to couple for 50 minutes at room temperature. For phosphorylated amino acids, coupling was extended to 1.5 hours. After the final deprotection step, peptides were washed twice with one syringe volume of dichloromethane (DCM) for 15 minutes at room temperature and placed in a vacuum desiccator for 30 minutes. Peptides were resuspended in a cleavage cocktail (95% trifluoroacetic acid (TFA), 2.5% triisopropyl silane, 2.5% water) and incubated at room temperature for 3 hours. The resulting peptide solution was concentrated under nitrogen and redissolved in a mixture of water/acetonitrile before purification by RP-HPLC.

**Peptide Purification.** Peptides were purified using an Agilent 1260 LC system equipped with an Agilent ZORBAX SB-C18 column (9.4 × 250 mm), 5 µm particle size employing water (A) and acetonitrile (B) mobile phase with 0.1% TFA. Fractions were characterized using matrix-assisted laser desorption/ionization time-of-flight (MALDI-TOF) mass spectrometry (Bruker) and Agilent 6530 quadrupole time-of-flight (Q-TOF) mass spectrometer coupled to an Agilent 1260 HPLC system. Pure fractions were combined and lyophilized and stored at -20 ºC as a 10 mM stock solution in water.

**Generation of Mutant Enzymes.** Point-mutated OspF genes were produced using QuikChange II Site-Directed Mutagenesis Kit (Agilent). Primers were designed using Agilent QuickChange Primer Design Tool and purchased from Invitrogen ThermoFisher. 2 µL of the PCR reaction were transformed into XL10-Gold ultracompetent (Agilent) *E. coli*. The following day, colonies were inoculated into 5 mL Luria Broth with 1:1000 ampicillin and grown at 37 ºC overnight (16-18 h). Cultures were pelleted at 4,700 RPM for 15 min, then mini-prepped using the QIAprep Spin Miniprep Kit. Extracted DNA was sequenced by Azenta GeneWiz and concentrations were determined using a Tecan Spark 10M microplate reader. OspF mutants were transformed into BL21-CodonPlusRIL (Agilent) *E. coli* and expressed as described in this paper. For ∆N26 mutant, gene was designed and purchased from GeneArt, then followed the above steps.

**Protein Expression and Purification.** Phospholyases were produced as described previously. Briefly, an *Escherichia coli* optimized copy containing OspF gene was cloned into the pRSETA vector, which fuses target proteins with an N-terminal His-tag. Expression vectors encoding OspF were transformed into BL21-CodonPlusRIL (Agilent) *E. coli*. Cultures were grown to an $OD_{600}$ of 0.4-0.6 at 37 °C, and expression was induced with 0.1 mM isopropyl β-D-1thiogalactopyranoside (IPTG) at 20 °C for 3-4 h. After expression, cells were pelleted and frozen. BugBuster protein extraction reagent was used along with 0.5 mM phenylmethanesulfonyl fluoride and Benzonase nuclease to lyse bacterial cells. Proteins were purified using the His-GraviTrap protein purification column eluting with elution buffer (500 mM imidazole, 100 mM Tris, and 50 mM NaCl (pH 7)). A buffer exchange was performed using Tris-buffered saline (100 mM Tris and 50 mM NaCl (pH 7)) to remove imidazole. The resulting pure protein samples were stored at −20 °C. Concentrations were determined using a Bio-Rad Bradford Protein Assay and a Tecan Spark 10M microplate reader.

**General Peptide Elimination Protocol.** Synthetic phosphopeptide elimination studies were conducted by incubating 1 mM peptide in TBS pH 8 at 26 ºC. Reaction was initiated upon the addition of 10 µM recombinant enzyme. To quench reactions, 5 µL of the sample was diluted in 200 µL 100 mM HCl.

**Peptide Study LC-MS Data Acquisition.** Reversed-phase chromatography and mass spectrometry were performed on an Agilent 1260 Infinity LC system in line with an Agilent 6530 Accurate Mass Q-TOF. Reversed-phase chromatography was performed on a ZORBAX 30SB-C8 column (2.1 mm x 100 mm, Agilent) using a water/acetonitrile gradient mobile phase containing 0.1% formic acid (0.4 mL/min; 2% ACN, isocratic 0-1.75 minutes, 2% - 48% 1.76-16 minutes). Data analysis was performed using Agilent MassHunter Qualitative Analysis software. Conversion from the phosphorylated parent peak to the eliminated product peak was calculated based on the detected peak volumes. Peak volumes (V) are determined using the Molecular Feature Extractor within the Agilent Qualitative Analysis software and represents ion counts observed for any and all charge states associated with a particular parent ion as previously described.[1–4] As peptides and their modified counterparts

can ionize differently (e.g. different charge states or different salt adducts) this method provides a more robust measure than comparing only a single charge state. A list of all peptides used and their m/z values are found in Table S1.

$$\%\ apparent\ conversion = 100 \times \frac{V_{product}}{V_{product} + V_{parent}} \tag{1}$$

**Cell Culture.** HeLa cells were purchased from ATCC (CCL-2) and grown in Dulbecco's Modified Eagle Medium supplemented with 10% (v/v) Fetal Bovine Serum and 10% (v/v) Penicillin-Streptomycin at 37 ºC with 5% $CO_2$. Cells were serum starved for 20 h and stimulated with 50 ng/mL recombinant EGF for 10 min prior to harvest. For anisomycin studies, cells were serum-starved for 20 h and stimulated with 25 µg/mL anisomycin in dimethyl sulfoxide for 30 min prior to harvest. Cells were washed with PBS and incubated with TrypLE Express for 5 minutes at 37 ºC. The resulting cell suspension was pelleted by centrifugation for 5 min at 200 x g. Pellets were washed with PBS and pelleted for 5 min at 200 x g for a total of two washes prior to lysis. Cells were lysed at 4 ºC in Pierce IP Lysis Buffer containing Halt protease and phosphatase inhibitor cocktail. Lysates were clarified by centrifugation at 13,500 rpm for 15 min at 4 ºC. Total protein content was determined by BCA. Aliquots were stored at -20 ºC.

***Shigella* Infection Assays.** HeLa cells (ATCC) were maintained in high glucose DMEM (11965118, Thermo Fisher Scientific) supplemented with 10% heat-inactivated fetal bovine serum (FBS, Atlanta Biologicals), 100 IU/mL penicillin, and 100 µg/mL streptomycin (Life Technologies). One day prior to infection, HeLa cells were seeded at $8x10^5$ cells/well in 6-well tissue culture treated plates (Corning) in antibiotic-free DMEM supplemented with 10% FBS. Single red colonies of wild-type or Δ*ospF* *Shigella flexneri* 2457T expressing AfaI[5] isolated from Congo red containing plates were inoculated into 2 mL TCS (trypticase soy) broth and culture at 30°C. The next day, each culture was diluted 1:50 into TCS broth and grown at 37°C until an $OD_{600}$ of 0.6 to 1.0. HeLa cells were than infected with *Shigella flexneri* resuspended in pre-warmed low-glucose DMEM supplemented with 1% FBS at an MOI (multiplicity of infection) of 10. Plates were centrifuged at 2,000 rpm for 10 min to synchronize the infection and incubated ° for 0.5 hr, after which each well was washed two times and then incubated in Hanks' Buffered Salt Solution, 10% FBS, 50 mM HEPES plus gentamicin (50 µg/mL). The plate was incubated for 0.5h, then the infected cells were treated with 0.5 ml TrypLE. After incubation for 5 min, TrypLE was inactivated by adding the same volume of DMEM supplemented with FBS (1%) and gentamicin (50 µg/mL). After centrifugation at 2,000 rpm for 5 min, the cells were washed with 1 mL PBS (phosphate-buffered saline), then re-centrifugated. The cells were lysed with the Pierce IP Lysis buffer containing Halt protease and phosphatase inhibitor cocktail. All mammalian cell incubations were conducted in a 5% $CO_2$ incubator at 37°C and all bacteria grown in test tubes on a roller.

**Chemoproteomics Studies.** Probe synthesis was performed as previously described.[2] To 2 mg HeLa lysate, TBS was added to bring lysate protein concentration to 1 µg/µL. Recombinant phospholyase was added to lysates and allowed 1 hour to incubate at 26 ºC to catalyze the formation of dehydrobutyrine in the proteome. After 1 hour, probe was added to a final concentration of 1 mM and allowed to incubate for 18 hours. Excess probe was removed using Pierce 3k MWCO Spin Concentrators. Briefly, 900 µL of TBS was added to probe-labeled lysates and then concentrated by centrifugation at 4000 x g for 1 h at room temperature. After 1 h, lysate was resuspended with 500 µL TBS and concentrated by centrifugation at 4000 x g for 1 h at room temperature and repeated once more. The volume of probe-labeled lysate was then brought to 1 mL and was added to 200 µL Pierce Streptavidin magnetic beads and incubated at 4 ºC overnight with end-over-end mixing. Beads were washed three times with TBS-T and eluted by heating to 96 ºC for 10 minutes in a solution of 3.3% sodium dodecyl sulfate (SDS), 200 mM DTT in Tris-HCl, pH 6.8. Eluted proteins were reduced with 5 mM DTT at 65 ºC for 15 minutes with 1,000 rpm shaking and alkylated in the dark with 20 mM iodoacetamide at room temperature for 30 minutes. To remove SDS, samples were applied to S-trap micro columns and prepared as described by the manufacturer. Proteins were digested on S-trap using 5 µg sequencing-grade trypsin in 50 mM triethylammonium bromide buffer at 37 ºC overnight. After eluting from S-trap columns, peptides were dried using a SpeedVac at 45 ºC for 1 h and stored at -20 ºC until use.

**Phospho-Protein Enrichment Studies.** For phosphoproteomics studies, all cells were lysed with Takara ProteoGuard™ EDTA-Free Protease Inhibitor Cocktail and supplemented with 10 mM sodium fluoride. HeLa lysate (200 µg) was applied to commercial ion-metal affinity chromatography (IMAC) magnetic resin (TALON® PMAC Magnetic Phospho Enrichment Kit) at 4 °C overnight and washed and eluted with buffers supplied by manufacturer to capture the phosphoproteome. The eluted proteins reduced as described above. Subsequently, the eluted phosphoproteins were applied to S-traps as described above and incubated with 5 µg sequencing grade trypsin overnight at 37 ºC. Peptides were dried using a SpeedVac at 45 ºC for 1 h and stored at -20 ºC until use.

**Whole Cell Lysate Proteomics Studies.** HeLa lysate (250 µg) was denatured in 4 M urea in 50 mM triethylammonium bicarbonate (TEAB) and reduced and alkylated as described above. SDS was added to a final concentration of 3.3%, and samples were applied to S-trap midi columns and processed as described by manufacturer. Proteins were digested on S-trap

using 1:100 w/w sequencing-grade trypsin in 50 mM triethylammonium bromide buffer at 37 ºC overnight. After eluting from S-trap columns, peptides were dried using a SpeedVac at 45 ºC for 1 hour and stored at -20 ºC until use.

**Proteomic Study LC-MS Data Acquisition.** Peptides were resuspended in 30 µL 0.1% formic acid in water and 2 µL of sample was injected onto a Vanquish Neo UHPLC system coupled with an Orbitrap Exploris 240 Mass Spectrometer for bottom-up proteomics with an EASY-Spray source interface between the LC and MS. Peptides were separated on EASY-Spray PepMap Neo columns (ES75750PN) with a maximum pressure set to 1500 bar. The system was controlled via Standard Instrument Integration for Xcalibur software. All hardware and data acquisition software were from Thermo Fisher Scientific. Mobile phase A was water with 0.1% formic acid (FA) and mobile phase B was 100% acetonitrile with 0.1% FA with all solvents being purchased from Thermo Fisher Scientific.

A 100 min linear gradient with a flow rate of 300 nL/min was used from 2-20%B, followed by a 40-minute linear gradient from 20-35%B, and finally a 10-minute gradient from 35-60%B. The autosampler was kept at a temperature of 4 ºC and column temperature maximum was set to 60 ºC. The Orbitrap Exploris MS was operated in data-dependent acquisition (DDA) mode using a full scan with an m/z range 350-1400 (resolution = 60,000, normalized AGC target = 300%, maximum injection time = 38 ms, dynamic exclusion for 100 s with a ±10 ppm window). The intensity threshold for precursors was 8.0e3. MS/MS spectra were acquired in DDA mode with a 3 s cycle time where each precursor above the intensity threshold was selected once before being added to the dynamic exclusion list for 100s with a +/- 10 ppm window and subsequently fragmented for MS2 analysis via higher-energy collisional dissociation (HCD) using a normalized collision energy (NCE) of 28% (resolution = 11500, normalized AGC target = 50%, the maximum injection time = 35ms).

**Western Blotting.** SDS loading dye (6X) containing dithiothreitol (DTT) was added to cellular samples and boiled at 95 ºC for 5 minutes. Samples were loaded into pre-cast 4-15% protein gels (mini-PROTEAN TGX, Bio-Rad) and electrophoresed for 30 minutes at 150 V. Proteins were transferred to a PVDF membrane using semi-dry transfer using an iBlot2 system (Invitrogen). Membranes were blocked for 1 hour at room temperature in 5% bovine serum albumin in TBS-T (20 mM Tris, 150 mM NaCl, and 0.1% Tween), and primary antibody was added for overnight incubation at 4 ºC with agitation. Blots were washed 3 times with TBS-T for 5 minutes at room temperature, and HRP-conjugated secondary antibody was added (1:2000 in BSA/TBS-T) for 50 minutes. After incubation with secondary antibody, blots were washed 3 times with TBS-T for 5 minutes at room temperature. Chemiluminescent signal was developed with Clarity Western ECL Substrate (Bio-Rad) and imaged using a Bio-Rad ChemiDoc XRS+. Antibody descriptions are found in Table S2.

**Proteomics Data Analysis**. All proteomics data was acquired with three biological replicates (n = 3). Acquired Orbitrap Exploris 240 .raw DDA files were converted to .mzML and processed using Fragpipe and searched against a human Uniprot protein database containing common contaminants using MS Fragger.[6,7] Database search criteria were as follows: fully tryptic with two missed cleavages, a precursor mass tolerance of 5 ppm and a fragment ion tolerance of 5 ppm. Static modifications included carboxyamidomethylation of cysteines (+57.0214 Da). Dynamic modifications for chemoproteomics included phosphorylation on serine, threonine, and tyrosine (+79.966), chemical modification with TCEP-biotin on threonine (+589.246 Da), oxidation of methionine (+15.9949 Da), and protein N-terminal acetylation (+42.0106 Da). Peptide-spectrum matches were adjusted to a 1% false discovery rate (FDR) using Percolator.[8] Label-free quantification was performed using IonQuant.[9] All bioinformatic analysis of LC-MS/MS data was performed in the R statistical computing environment as previously described. Upregulation was defined as a protein that exhibited a log2FC ≥ 1.0 and a p-value ≤ 0.05. Proportional Venn diagrams were using https://www.deepvenn.com/.

**Proteomics Data Availability.** The mass spectrometry proteomics data have been deposited to the ProteomeXchange Consortium via the PRIDE partner repository with the dataset identifier PXD073985.

| **Peptide Number** | **7-mer Sequence** | **Expected m/z** | **Observed m/z** |
|---|---|---|---|
| **1** | GFLpTEYV-$NH_2$ | 907.40 | 907.40 |
| **2** | YFMpTEYV-$NH_2$ | 1031.36 | 1031.39 |
| **3** | QAVpTEYV-$NH_2$ | 888.35 | 888.39 |
| **4** | FMMpTPYV-$NH_2$ | 966.37 | 966.37 |
| **5** | GFLpTEKV-$NH_2$ | 872.42 | 872.42 |
| **6** | VDYpTTAK-$NH_2$ | 925.42 | 925.41 |
| **7** | FNLpTGLN-$NH_2$ | 857.39 | 857.39 |
| **8** | NFKpSPVK-$NH_2$ | 897.45 | 897.45 |
| **9** | LAEpTYHL-$NH_2$ | 876.39 | 876.38 |
| **10** | ELQpTIGF-$NH_2$ | 886.40 | 886.40 |
| **11** | KLKpTQML-$NH_2$ | 940.49 | 940.50 |
| **12** | DALpSGSG-$NH_2$ | 685.25 | 685.25 |
| **13** | DNFpSLHD-$NH_2$ | 926.34 | 926.33 |
| **14** | AFVpTFDD-$NH_2$ | 893.34 | 893.34 |

**Table S1. Peptide masses.** Peptides synthesized for this study with their expected and observed m/z values.

| Antibody | Vendor (Product Number) | Host Species | Dilution |
|---|---|---|---|
| **ERK1/2** | Cell Signaling Technologies (9102) | Rabbit | 1:2000 |
| **pERK1/2** | Cell Signaling Technologies (9106) | Mouse | 1:2000 |
| **JNK** | Cell Signaling Technologies (9252) | Rabbit | 1:2000 |
| **pJNK** | Cell Signaling Technologies (9251) | Rabbit | 1:2000 |
| **RAB1A** | Thermo Fisher (A305-328A) | Rabbit | 1:2000 |
| **CSNK2B** | Thermo Fisher (A301-983A) | Rabbit | 1:2000 |
| **pCSNK2B** | Thermo Fisher (44-1090G) | Rabbit | 1:1000 |
| **DDX6** | Thermo Fisher (14632-1-AP) | Rabbit | 1:1000 |
| **UGDH** | ProteinTech (67360-1-Ig) | Mouse | 1:1000 |
| **Biotin-HRP** | Cell Signaling Technologies (7075) | Rabbit | 1:2000 |
| **Mouse-HRP** | Cell Signaling Technologies (7076) | Horse | 1:2000 |
| **Rabbit-HRP** | Cell Signaling Technologies (7074) | Goat | 1:2000 |

**Table S2. Antibodies.** Antibodies used in this study.

| Gene | UniProt Accession |
|---|---|
| RAB1A | P62820 |
| DDX6 | P26196 |
| LGALS3 | P17931 |
| CSNK2B | P67870 |
| KIF5B | P33176 |
| HNRNPA1 | P09651 |
| UGDH | O60701 |
| FLNC | Q14315 |
| AGO1 | Q9UL18 |
| DAZAP1 | Q96EP5 |
| ATXN1 | P54253 |
| NCOA3 | Q9Y6Q9 |
| DOCK7 | Q96N67 |
| PLXNB2 | O15031 |
| TSN | Q15631 |
| COA7 | Q96BR5 |
| MTHFD1L | Q6UB35 |
| XXYLT1 | Q8NBI6 |
| DNAJC15 | Q9Y5T4 |
| UTP11 | Q9Y3A2 |
| PIK3C3 | Q8NEB9 |
| EIF2AK2 | P19525 |
| CSNK1A1 | P48729 |
| NEK7 | Q8TDX7 |
| CSNK1D | P48730 |
| TP53RK | Q96S44 |
| WNK1 | Q9H4A3 |
| CDK9 | P50750 |
| ILK | Q13418 |
| TRIO | Q9Y4E5 |
| MAP2K3 | P46734 |
| TRIM28 | Q13263 |
| SRPK1 | Q96SB4 |
| RPS6KA4 | O75676 |
| CDK1 | P06493 |
| MARK3 | Q99638 |
| MARK2 | Q7KZI7 |
| PRKDC | P78527 |
| VRK1 | Q99986 |

**Table S3. UniProt accession information.** Identifiers for proteins experimentally identified in this study.

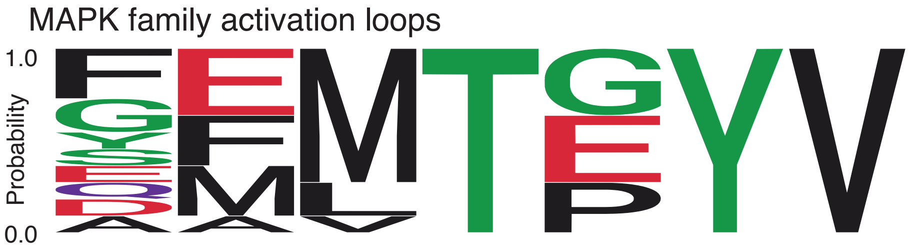


| MAPK Gene | UniProt ID | Common Name | Activation Loop (-10/X/+10) |
|---|---|---|---|
| **MAPK1** | P28482 | ERK2 | DPDHDHTGFL**pT**EYVATRWYRA |
| **MAPK3** | P27361 | ERK1 | DPEHDHTGFL**pT**EYVATRWYRA |
| **MAPK4** | P31152 | ERK4 | DQHYSHKGYL**pS**EGLVTKWYRS |
| **MAPK6** | Q16659 | ERK3 | DPHYSHKGHL**pS**EGLVTKWYRS |
| **MAPK7** | Q13164 | ERK5 | TSPAEHQYFM**pT**EYVATRWYRA |
| **MAPK8** | P45983 | JNK1 | ARTAGTSFMM**pT**PYVVTRYYRA |
| **MAPK9** | P45984 | JNK2 | ARTACTNFMM**pT**PYVVTRYYRA |
| **MAPK10** | P53779 | JNK3 | ARTAGTSFMM**pT**PYVVTRYYRA |
| **MAPK11** | Q15759 | p38 beta | GLARQADEEM**pT**GYVATRWYRA |
| **MAPK12** | P53778 | p38 gamma | GLARQADSEM**pT**GYVVTRWYRA |
| **MAPK13** | O15264 | p38 delta | GLARHADAEM**pT**GYVVTRWYRA |
| **MAPK14** | Q16539 | p38 alpha | GLARHTDDEM**pT**GYVATRWYRA |
| **MAPK15** | Q8TD08 | ERK7/8 | LPEGPEDQAV**pT**EYVATRWYRA |

**Figure S1. MAPK activation loops.** Consensus sequence for the activation loops for all 14 MAPK family members. Corresponding table containing sequence data for human MAPK activation loops.

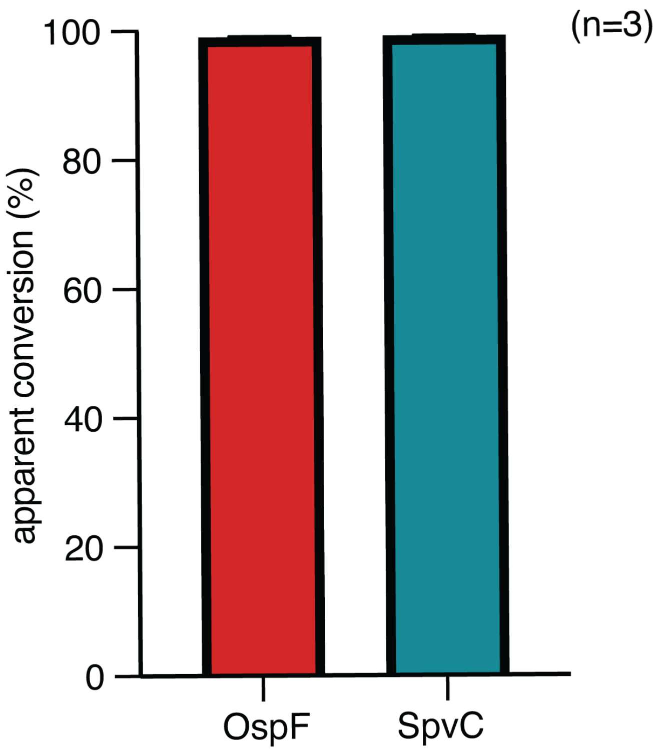


**Figure S2. Overnight incubation of pJNK peptide with phospholyases.** Peptide **4** was incubated with either OspF or SpvC for 24 hours. Apparent conversion was monitored by LC-MS and revealed that both enzymes eventually converted all phosphopeptide to corresponding Dhb species.

```
OspF WT  MPIKKPCLKLNLDSLNVVRSEIPQMLSANERLKNNFNILYNQIRQYPAYYFKVASNVPTY  60
SpvC WT  MPINRPNLNLNIPPLNIVAAYDGAEIPSTNKHLKNNFNSLHNQMRKMPVSHFKEALDVPD

OspF WT  SDICQSFSVMYQGFQIVNHSGDVFIHACRENPQSKGDFVGDKFHISIAREQVPLAFQILS  120
SpvC WT  YSGMRQSGFFAMSQGFQLNNHGYDVFIHARRESPQSQGKFAGDKFHISVLRDMVPQAFQA

OspF WT  GLLFSEDSPIDKWKITDMNRVSQQSRVGIGAQFTLYVKSDQECSQYSALLLHKIRQFIMC  180
SpvC WT  LSGLLFSEDSPVDKWKVTDMEKVVQQARVSLGAQFTLYIKPDQENSQYSASFLHKTRQFI

OspF WT  LESNLLRSKIAPGEYPASDVRPEDWKYVSYRNELRSDRDGSERQEQMLREEPFYRLMIE  239
SpvC WT  ECLESRLSENGVISGQCPESDVHPENWKYLSYRNELRSGRDGGEMQRQALREEPFYRLMTE  241
```

**Figure S3. OspF and SpvC sequence alignment.** Sequence alignment of phospholyases OspF and SpvC based on UnitProt entries Q8VSP9 (OspF) and P0A2M9 (SpvC).

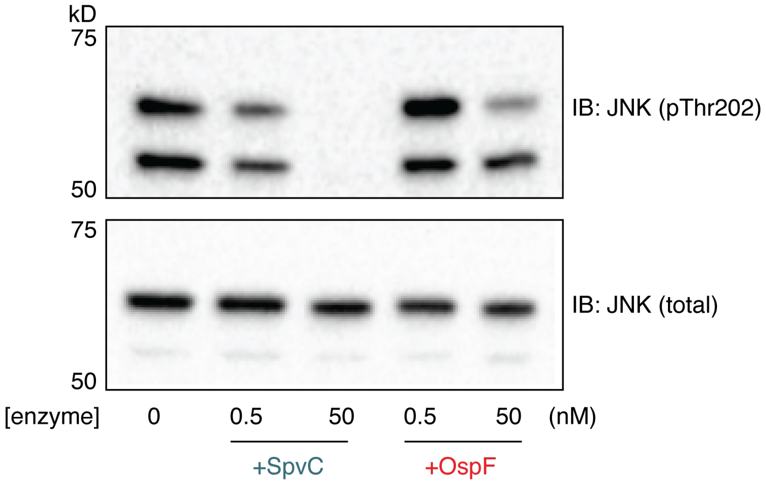


**Figure S4. Western blots of pJNK/total JNK.** HeLa lysates were stimulated with anisomycin, harvested, lysed, incubated with recombinant phospholyase at various concentrations for 1 h at 26 °C, and run on a western blot to probe for pJNK and total JNK. Some elimination of pJNK can be seen with 0.5 nM SpvC, and complete elimination can be seen with 50 nM. No elimination can be seen with 0.5 nM OspF, and some elimination with 50 nM OspF.

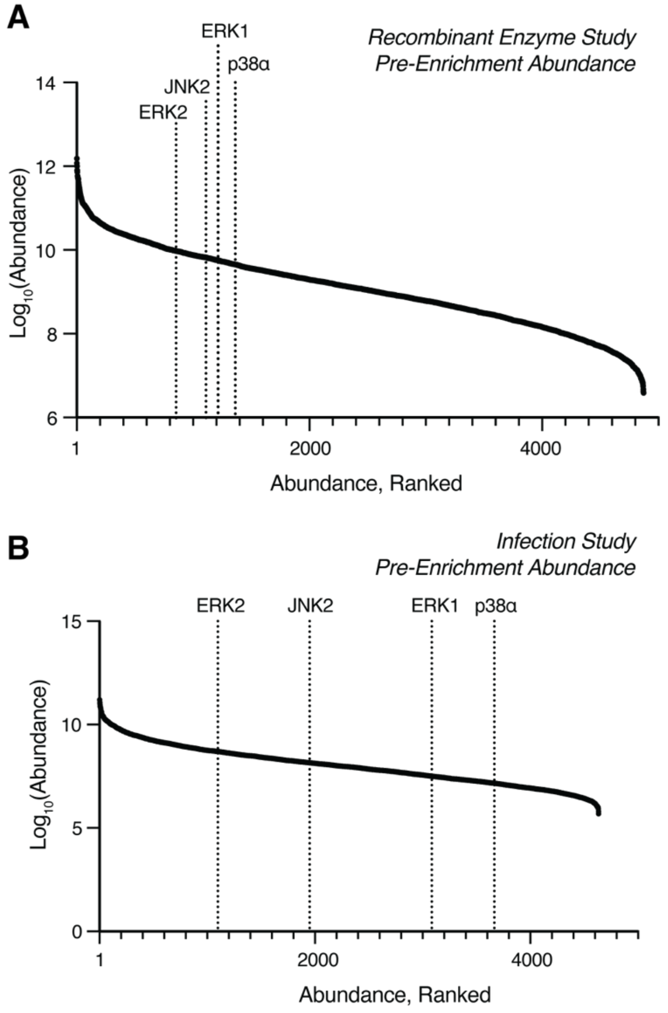


**Figure S5. Protein abundance for HeLa lysate after EGF stimulation and bacterial infection.** To monitor overall trends in protein expression by LCMS, we digested HeLa lysate either stimulated with EGF (A) or infected with *Shigella flexneri* (B) and calculated and ranked protein abundance.

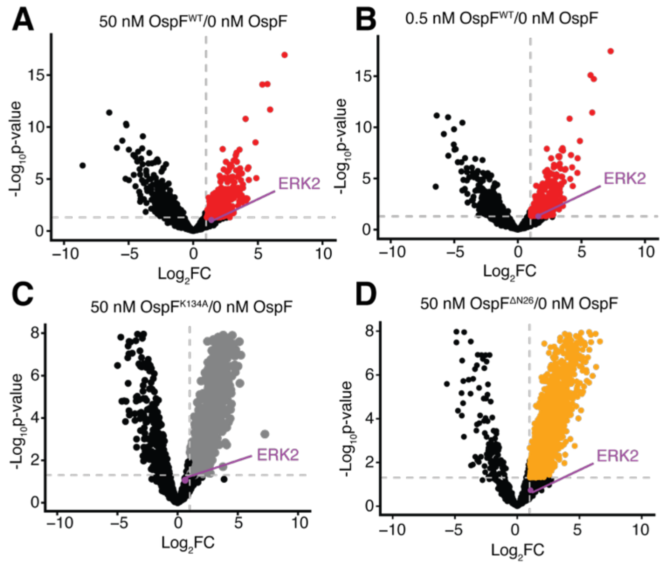


**Figure S6. Volcano plots compared to 0 nM OspF.** Volcano plots for proteins pulled down from EGF-stimulated HeLa lysate using probe **1** after treatment with 50 nM OspF$^{WT}$ (A), 0.5 nM OspF$^{WT}$ (B), 50 nM OspF$^{K134A}$ (C), and 50 nM OspF$^{\Delta N26}$ (D) relative to untreated EGF-stimulated HeLa lysate.

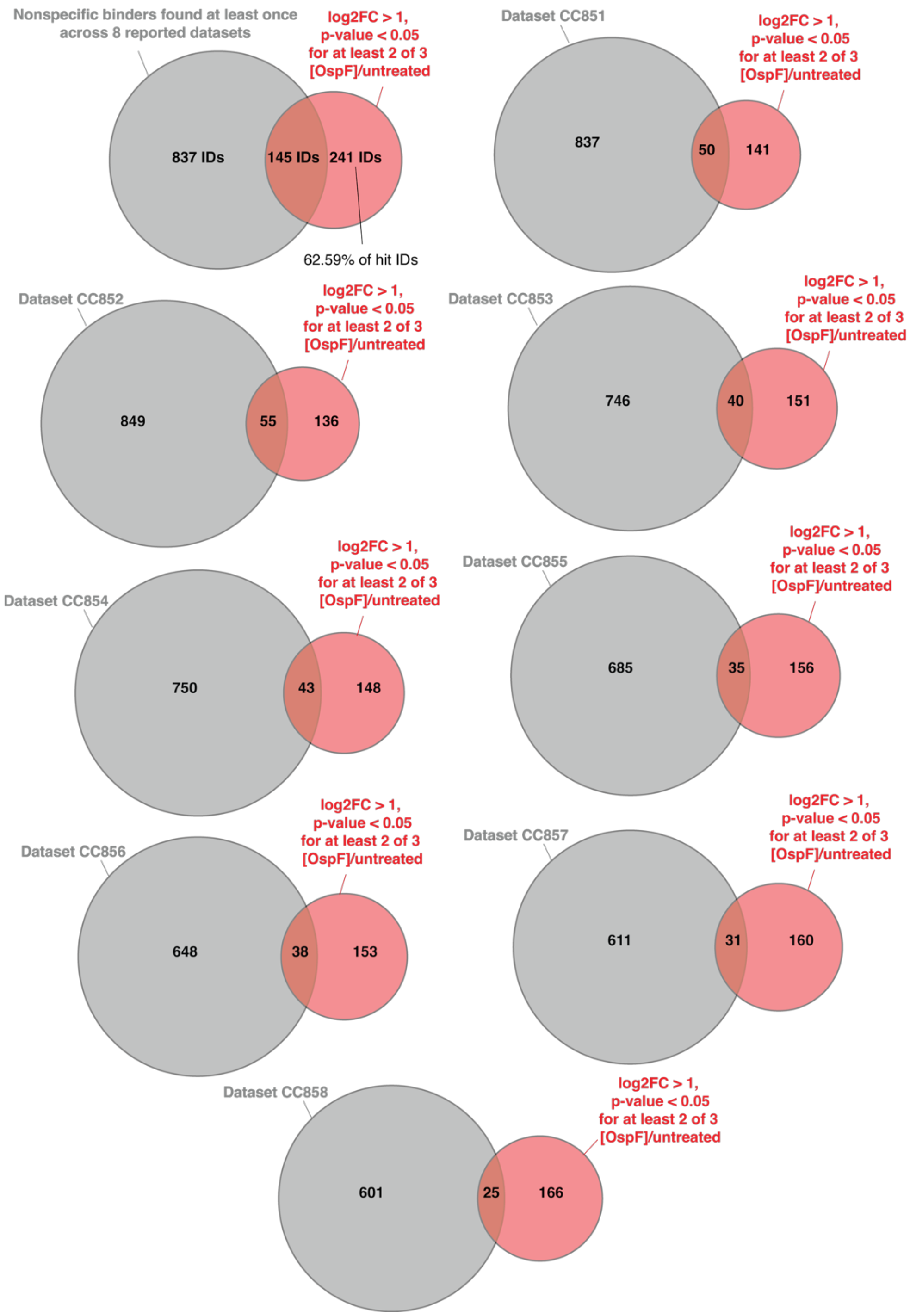


**Figure S7. Venn diagram for nonspecific binders.** As a qualitative assessment of what proportion of proteins enriched at a minimum 2 of 3 concentrations of $OspF^{WT}$ are possible nonspecific streptavidin binders, we queried a repository for nonspecific binders of streptavidin affinity supports.[10] Consistently, our hits were largely specific per each individual experimental list of nonspecific binders, and over 60% of our hits were specific using extremely stringent criteria for nonspecific binding.

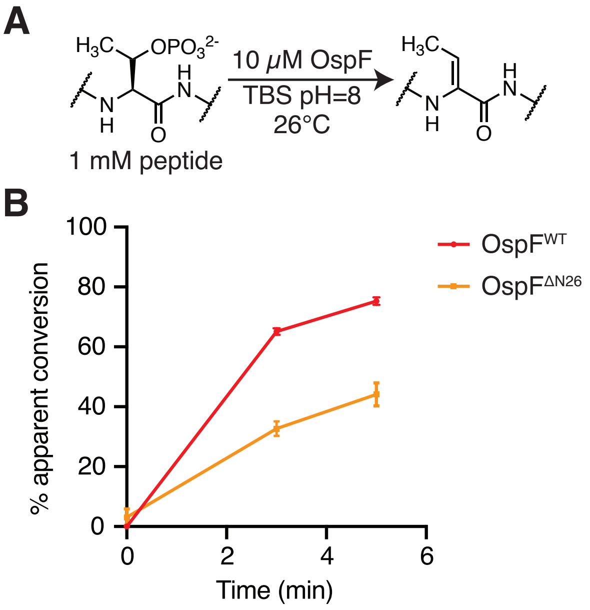


**Figure S8. Elimination of peptide 1 between OspF$^{WT}$ and OspF$^{\Delta N26}$**. (A) Reaction scheme to show OspF-elimination on phosphopeptides using LC-MS. (B) Time course showing conversion of peptide **1** to peptide **1$^{Dhb}$** between OspF$^{WT}$ and OspF$^{\Delta N26}$. Conversion is plotted as means (n = 3) ± standard error (SE).

$$\hat{f}(x) = KDE(\{log2\left(\frac{OspF_{WT,i}}{OspF_{\Delta N26,i}}\right)\}_{i \in \mathbb{P}}$$

$$Where\ the\ protein\ set\ \mathbb{P} = \{i: log_2(\frac{OspF_{wt,i}}{untreated_i}) > 0 \wedge log_2(\frac{OspF_{wt,i}}{OspF_{K134A,i}}) > 0\} \quad (2)$$

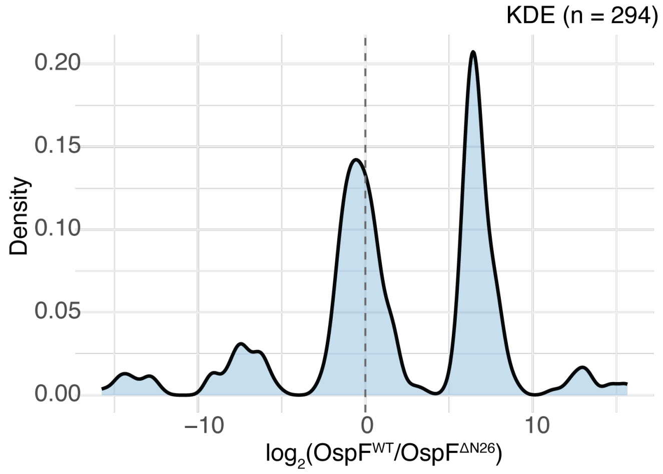


**Figure S9. Kernel density estimate (KDE) between OspF$^{WT}$ and OspF$^{\Delta N26}$.** (Top) Formula for KDE calculation for protein abundances. (Bottom) KDE plot for protein abundances quantified in EGF-stimulated HeLa lysate after pulldown with 50 nM OspF$^{WT}$ that pass filters for enrichment relative to untreated and catalytically dead (OspF$^{K134A}$) conditions. Using the top equation, these protein abundances were fit the KDE plot relative to their quantifiable abundances after pulldown from lysates treated with 50 nM OspF$^{\Delta N26}$.

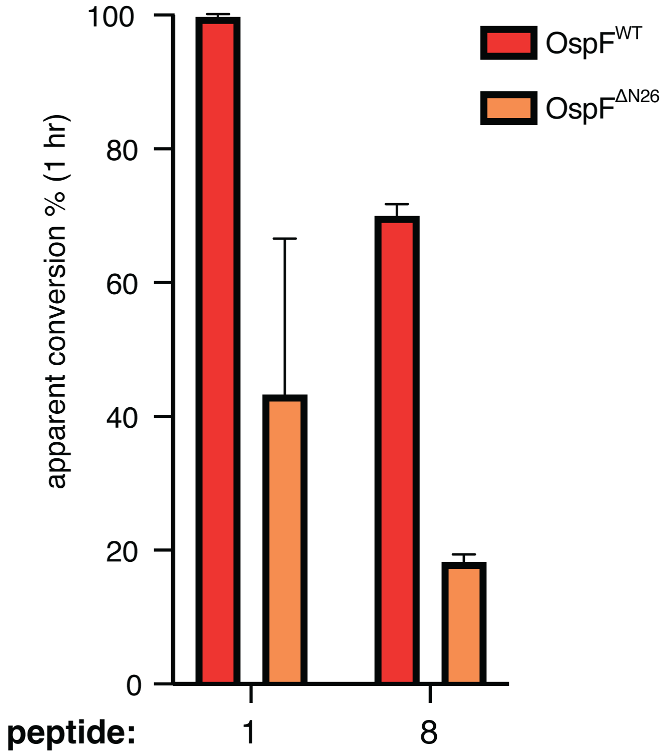


**Figure S10. Elimination of peptide 1 and 8 between OspF$^{WT}$ and OspF$^{\Delta N26}$.** Bar plot depicting conversion of peptide **1** (GFLpTEYV-$NH_2$) and peptide **8** (LAEpTHYL-$NH_2$) to corresponding Dhb species after 1 hour incubation with either OspF$^{WT}$ or OspF$^{\Delta N26}$ under conditions described in **Figure S5**. Conversion is plotted as means (n = 3) ± standard error (SE).

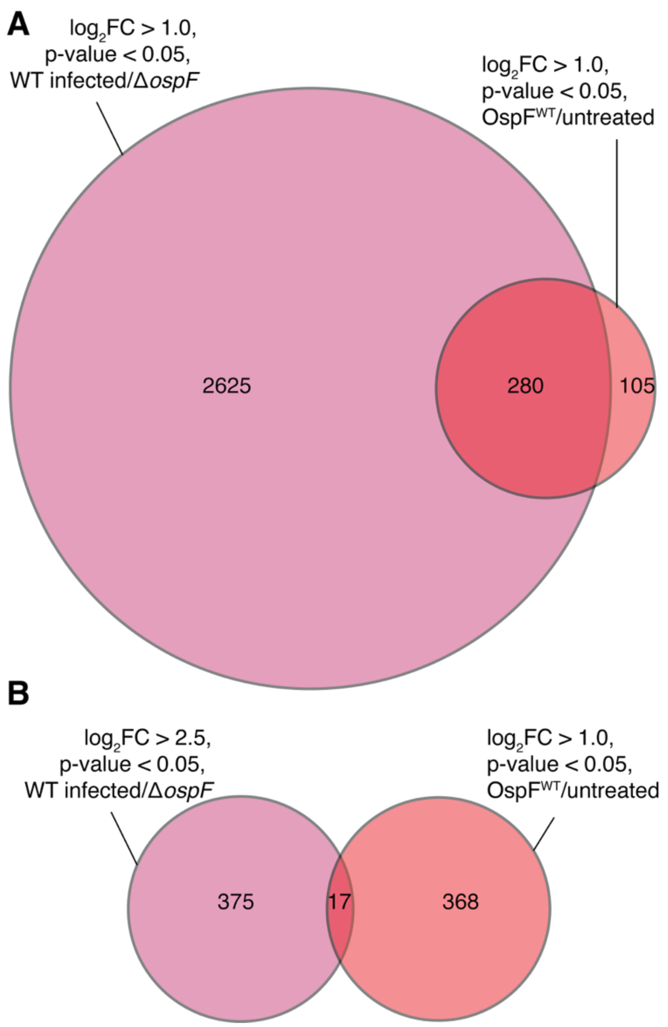


**Figure S11. Upregulated proteins after recombinant and *Shigella*-delivered OspF exposure.** Venn diagrams depicting the overlap between pulled down proteins after treatment with probe **1** found in our infection study and proteins pulled down after probe **1** treatment for at least 2 of 3 OspF concentrations in our *in vitro* study using either standard (A) or stringent (B) $\log_2$FC thresholds.

| Cluster | Name | Enrichment Score |
|---|---|---|
| **1** | Nucleus/Nucleoplasm Localization | 9.75 |
| **2** | Cytoplasm/Cytosol Localization | 9.05 |
| **3** | ATP/Nucleotide Binding & Kinase Activity | 6.1 |
| **4** | rRNA Processing & Ribosome Biogenesis | 5.71 |
| **5** | Endoplasmic Reticulum Localization | 5.67 |
| **6** | Mitochondrion Localization & Transit Peptide | 4.55 |
| **7** | Ubiquitin-like Conjugation & Isopeptide Bond | 3.68 |
| **8** | Mitochondrion Inner Membrane/Intermembrane | 3.46 |
| **9** | Glycosyltransferase Activity & N-linked Glycosylation | 3.07 |
| **10** | Mitochondrial Respiratory Chain & Neurodegeneration Pathways | 2.72 |
| **11** | DNA Damage Response & Repair | 2.68 |
| **12** | Chromosome/Telomere & DNA Replication | 2.57 |
| **13** | Transferase/Kinase Activity | 2.2 |
| **14** | Lipid Metabolism & Biosynthesis | 2.13 |
| **15** | Cell Junctions & Focal Adhesion | 2.04 |
| **16** | ABC Transporters & ATPase Activity | 1.98 |
| **17** | Protein Transport | 1.97 |
| **18** | Ribosomal Small Subunit Biogenesis | 1.95 |
| **19** | N-Glycan Biosynthesis & Glycosylation | 1.8 |
| **20** | ER-Golgi Vesicle-Mediated Transport | 1.72 |
| **21** | MATH/TRAF Domain Proteins | 1.64 |
| **22** | Phosphatidylinositol Signaling & Kinase Domains | 1.6 |
| **23** | DNA Replication Initiation | 1.6 |
| **24** | HECT E3 Ubiquitin Ligase & Ubiquitination | 1.56 |
| **25** | Translation Initiation & Protein Biosynthesis | 1.55 |
| **26** | Phospholipid Biosynthesis & Metabolism | 1.55 |
| **27** | Cell Cycle & Division | 1.51 |
| **28** | RNA Polymerase II Transcription Regulation | 1.46 |
| **29** | TPR Repeat Proteins | 1.4 |
| **30** | WD40 Repeat Proteins | 1.34 |

**Table S4. Functional enrichment clusters.** Names and enrichment scores given to statistically significant clusters of ontological terms as determined by DAVID (Supplemental Excel File 2). Statistical significance was calculated by DAVID as the geometric mean of EASE score with $-\log_{10}$ transformation.

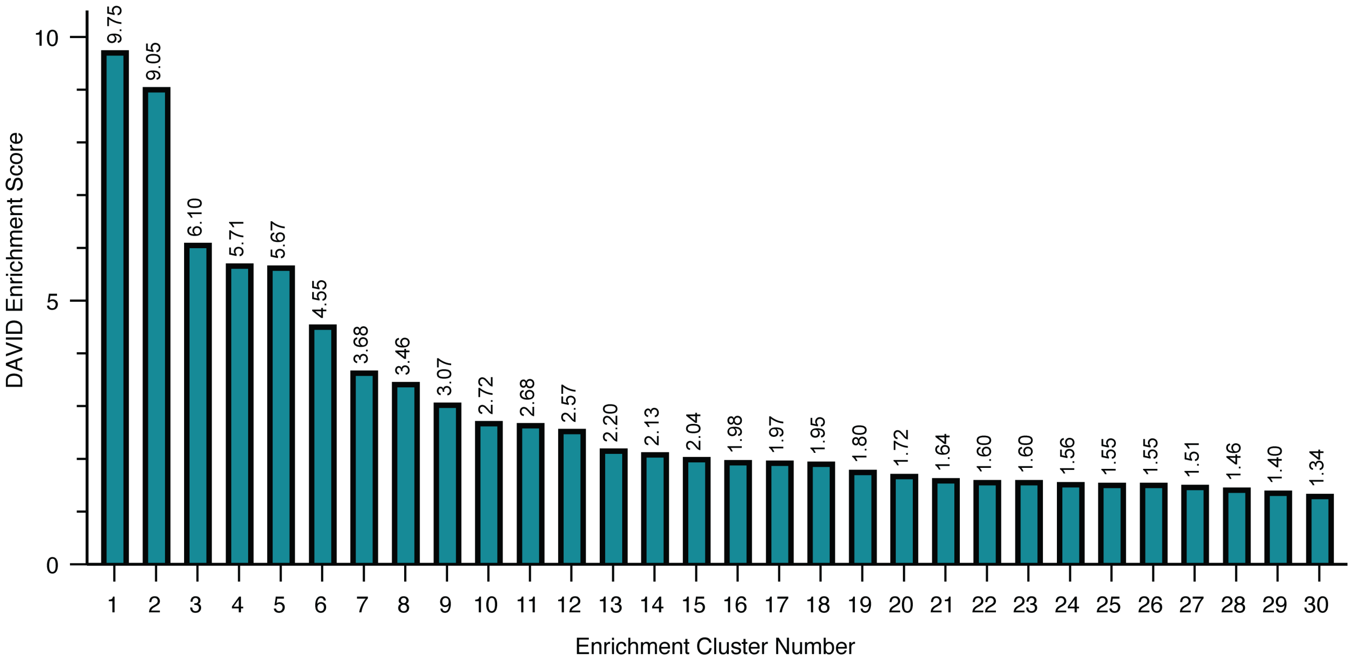


**Figure S12. Score distribution for enrichment clusters.** Bar plot depicting statistically significant enrichment clusters (Table S4) distributed from highest to lowest enrichment score.

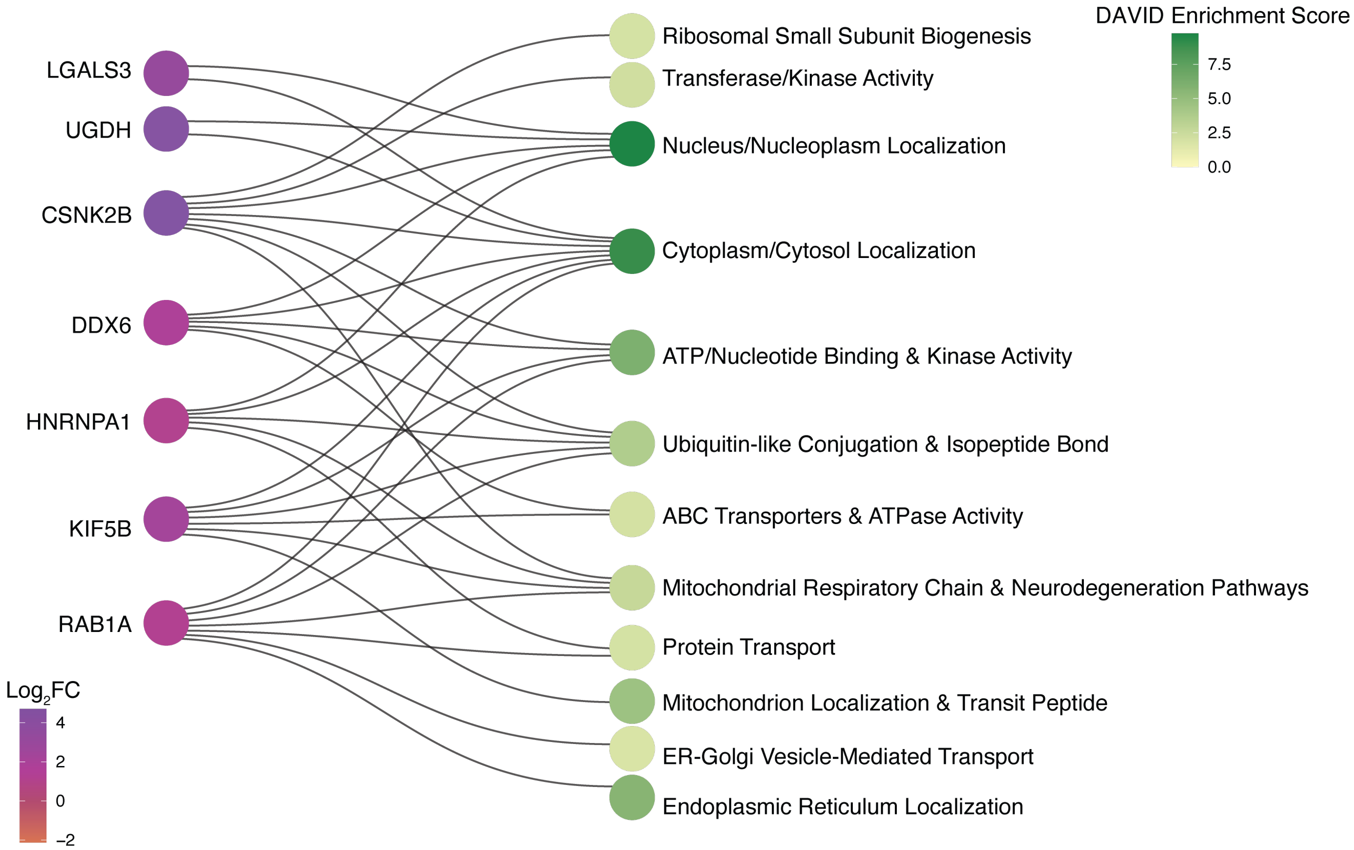


**Figure S13. Functional enrichment clustering contexts for genes followed up during peptide studies.** Alluvial/Sankey-type diagram depicting gene names of hits (Figure 6A) as nodes connected to clusters (Table S4). Gene nodes are colored by $\log_2$FC (p-value < 0.05) relative to WT/Δ*ospF Shigella* infection as shown in Figure 5B. Cluster nodes are colored by DAVID enrichment score as shown in Table S4. Diagram adapted from sankeymatic.com.

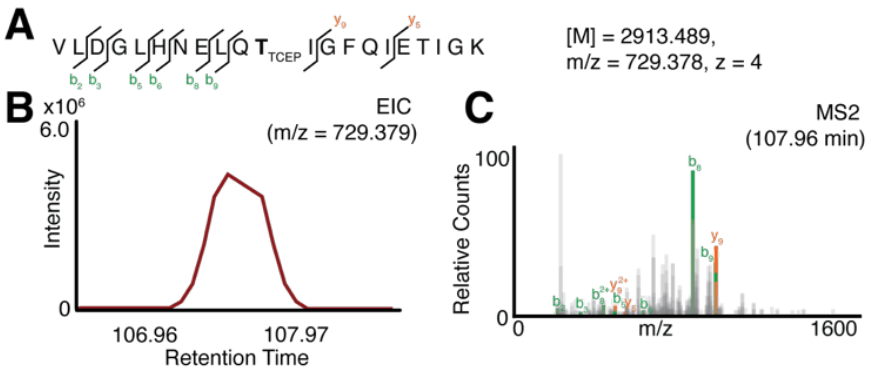


**Figure S14. Representative EIC and MS2 spectrum for UGDH$^{TCEP}$.** (A) Tryptic fragment and calculated mass [M] and m/z for UGDH$_{TCEP}$ in HeLa lysate infected with WT *Shigella* after treatment with probe **1** and pulldown. (B) Extracted ion chromatogram (EIC) with Gaussian smoothing for the above tryptic fragment with observed m/z. (C) MS$^2$ spectrum from this tryptic fragment displaying b and y ion coverage, accessed via Fragpipe's PDV viewer.

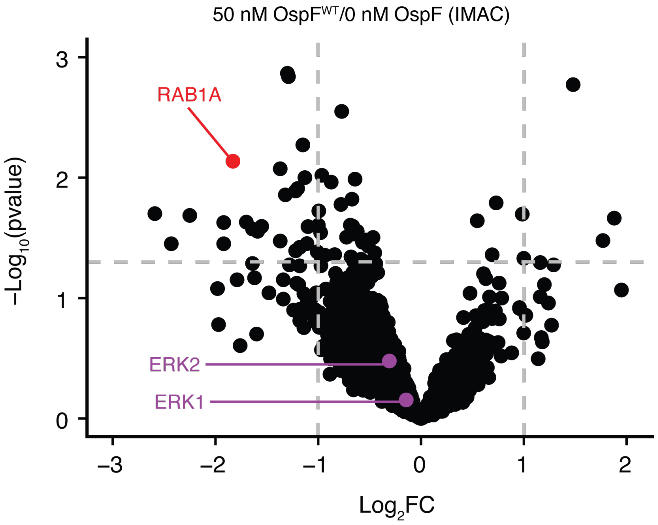


**Figure S15. Volcano plot for 50 nM OspF and IMAC.** Volcano plot depicting proteins upregulated ($log_2FC > 1$, p-value < 0.05) and downregulated ($log_2FC < -1$ , p-value < 0.05) after treatment with ±50 nM OspF$^{WT}$ after enrichment using IMAC resin.

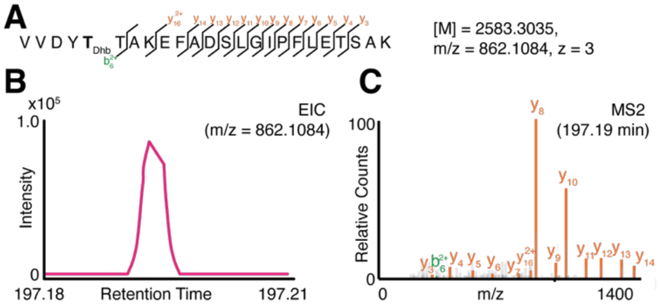


**Figure S16. Representative EIC and MS$^2$ spectrum for RAB1A$^{Dhb}$.** (A) Tryptic fragment and calculated mass [M] and m/z for RAB1A$^{Dhb}$ as observed in un-enriched lysate in the absence of probe after infection with WT *Shigella*. (B) Extracted ion chromatogram (EIC) with Gaussian smoothing for the above tryptic fragment with observed m/z. (C) MS$^2$ spectrum from this tryptic fragment displaying b and y ion coverage, accessed via Fragpipe's PDV viewer.[11]

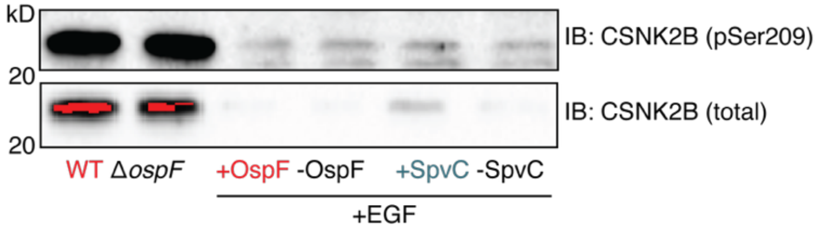


**Figure S17. Western blots of pCSNK2B/total CSNK2B.** For the left two lanes, lysate infected with *Shigella* were run. For the right four lanes, HeLa lysates were stimulated with EGF, harvested, lysed, incubated with 50 nM phospholyase for 1 hour at 26°C in TBS buffer (pH = 8), and run on a western blot to probe for pCSNK2B and total CSNK2B. Exposure to phospholyase through *Shigella* infection and through incubation with recombinant OspF and SpvC revealed no distinguishable changes to pSer209 phosphorylation in the absence of phospholyase.

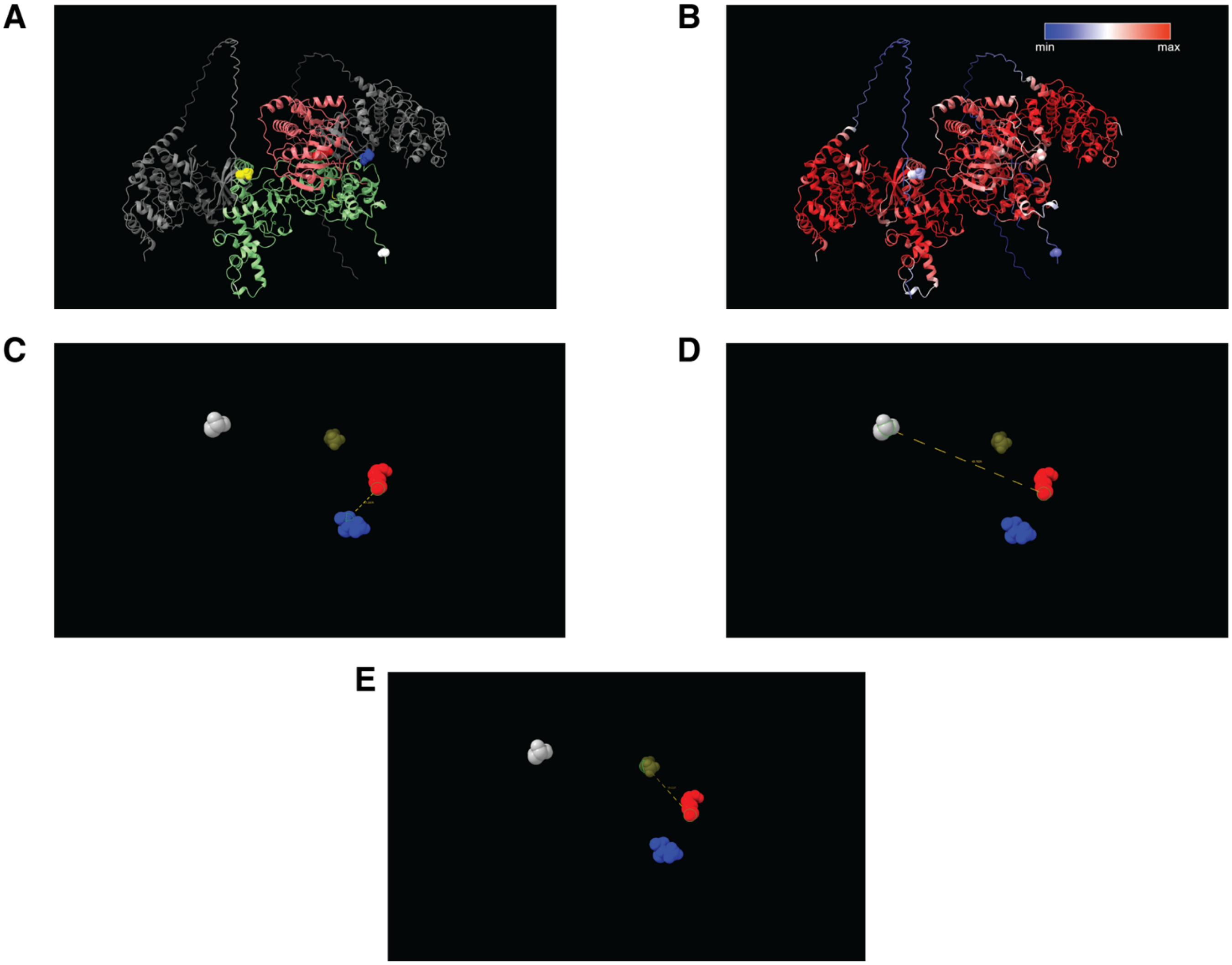


**Figure S18. AlphaFold images of OspF with CSNK2A/B.** (A) A depiction of OspF in complex with the CSNK2 tetramer. (CSNK2A1/CSNK2A1/CSNK2B/CSNK2B) as predicted by AlphaFold3 in the presence of $Mg^{2+}$ with phosphorylation input at Ser2 (white), Thr37 (blue), and Ser209 (yellow) on CSNK2B. CSNK2A1 subunits are colored in gray, CSNK2B subunits colored in green, and OspF colored in light red (Lys134 colored in red).[12] For this prediction, ipTM = 0.58 and pTM = 0.63. Cartoons accessed via ChimeraX.[13] (B) OspF-CSNK2 complex colored by pIDDT confidence. (C) CSNK2B-pThr37 depicted in isolation with OspF-Lys134 and other phospho-sites with a yellow line extending between the Lys134-Nϵ and pThr37-Cα, with distance between Lys134 and the Cα of CSNK2B-pSer2 and CSNK2B-pSer209 depicted in (D) and (E) respectively.

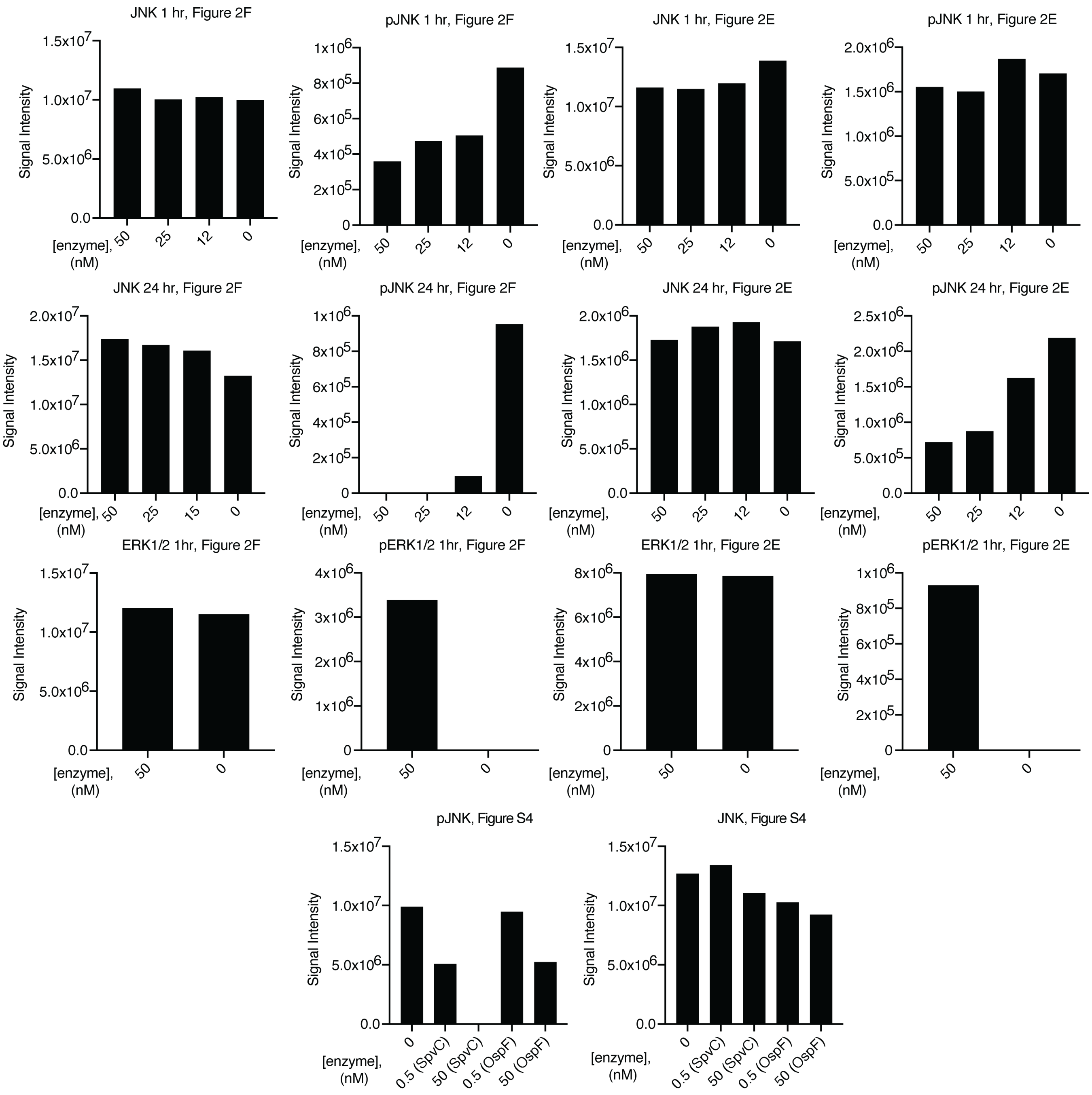


**Figure S19. Densitometry for western blots from JNK study.** We quantified bands using BioRad Image Lab 6.0.1. to numerically measure chemiluminescence for representative western blots associated with our JNK study.

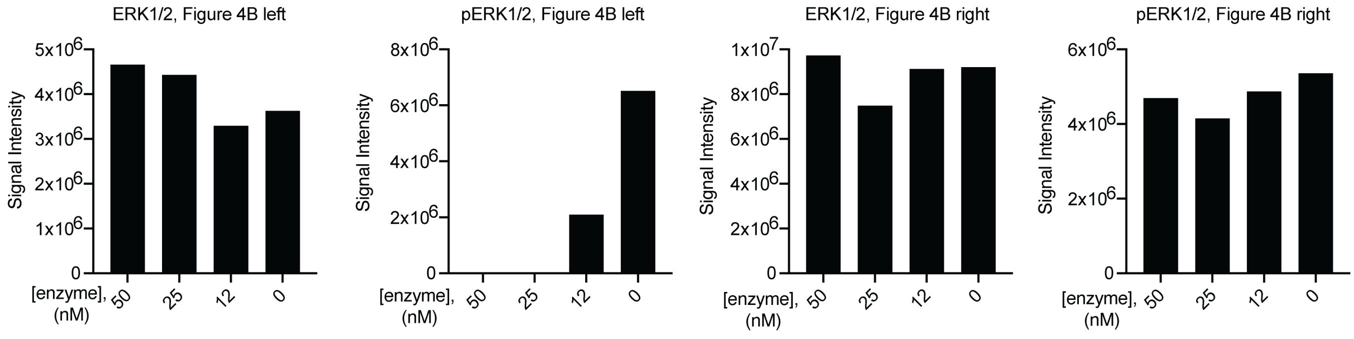


**Figure S20. Densitometry for western blots from D-domain study.** We quantified bands using BioRad Image Lab 6.0.1. to numerically measure chemiluminescence for representative western blots associated with study of OspF$^{\Delta N26}$.

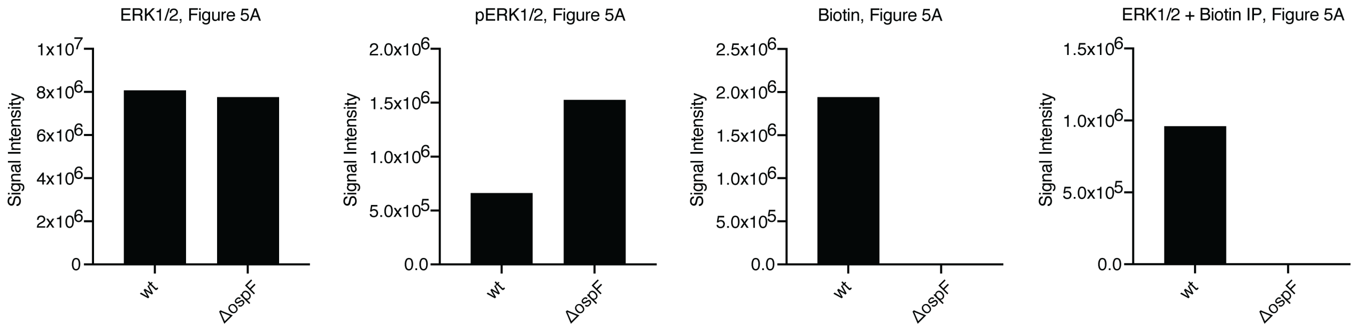


**Figure S21. Densitometry for western blots from infection chemoproteomics study.** We quantified bands using BioRad Image Lab 6.0.1. to numerically measure chemiluminescence for representative western blots associated with detection of ERK1/2 and modified ERK1/2 after *Shigella flexneri* infection.

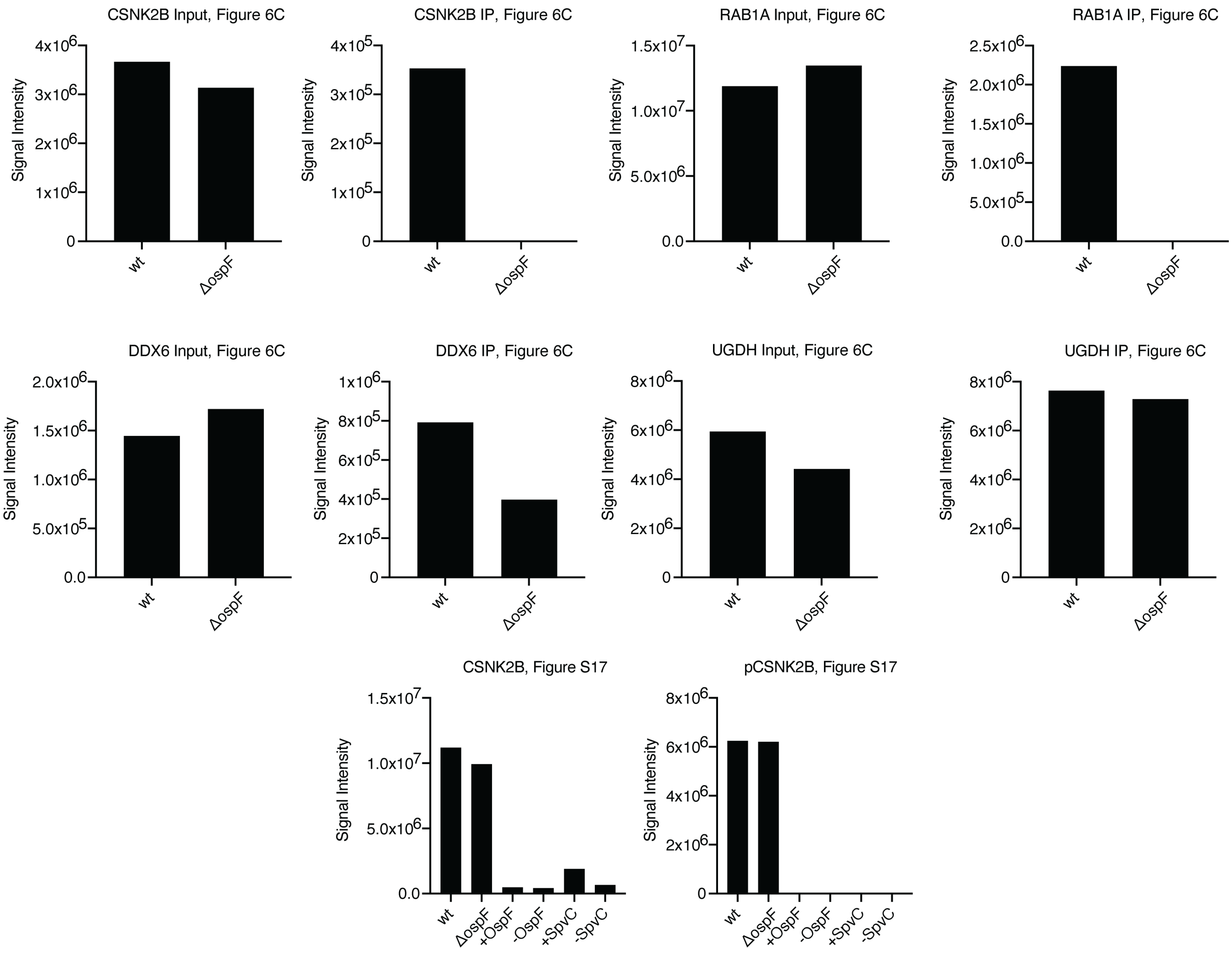


**Figure S22. Densitometry for western blots from hit validation study.** We quantified bands using BioRad Image Lab 6.0.1. to numerically measure chemiluminescence for representative western blots associated with protein-level validation of our hits reported in Figure 6.

**References:**


(1) Chambers, K. A.; Abularrage, N. S.; Scheck, R. A. Selectivity within a Family of Bacterial Phosphothreonine Lyases. *Biochemistry* **2018**, *57* (26), 3790–3796. https://doi.org/10.1021/acs.biochem.8b00534.

(2) Chambers, K. A.; Abularrage, N. S.; Hill, C. J.; Khan, I. H.; Scheck, R. A. A Chemical Probe for Dehydrobutyrine. *Angew. Chem. Int. Ed.* **2020**, *59* (19), 7350–7355. https://doi.org/10.1002/anie.202003631.

(3) McEwen, J. M.; Fraser, S.; Guir, A. L. S.; Dave, J.; Scheck, R. A. Synergistic Sequence Contributions Bias Glycation Outcomes. *Nat. Commun.* **2021**, *12* (1), 3316. https://doi.org/10.1038/s41467-021-23625-8.

(4) Tin Pham, V. T.; Datta, S.; Sterling, A. C.; Hansel, S. M.; Scheck, R. A. A Chemical Mechanistic Path Leads the Way to Cellular Argpyrimidine. *J. Am. Chem. Soc.* **2025**, *147* (42), 38055–38068. https://doi.org/10.1021/jacs.5c09369.

(5) Clerc, P.; Sansonetti, P. J. Entry of Shigella Flexneri into HeLa Cells: Evidence for Directed Phagocytosis Involving Actin Polymerization and Myosin Accumulation. *Infect. Immun.* **1987**, *55* (11), 2681–2688. https://doi.org/10.1128/iai.55.11.2681-2688.1987.

(6) Kong, A. T.; Leprevost, F. V.; Avtonomov, D. M.; Mellacheruvu, D.; Nesvizhskii, A. I. MSFragger: Ultrafast and Comprehensive Peptide Identification in Mass Spectrometry–Based Proteomics. *Nat. Methods* **2017**, *14* (5), 513–520. https://doi.org/10.1038/nmeth.4256.

(7) Teo, G. C.; Polasky, D. A.; Yu, F.; Nesvizhskii, A. I. Fast Deisotoping Algorithm and Its Implementation in the MSFragger Search Engine. *J. Proteome Res.* **2021**, *20* (1), 498–505. https://doi.org/10.1021/acs.jproteome.0c00544.

(8) Käll, L.; Canterbury, J. D.; Weston, J.; Noble, W. S.; MacCoss, M. J. Semi-Supervised Learning for Peptide Identification from Shotgun Proteomics Datasets. *Nat. Methods* **2007**, *4* (11), 923–925. https://doi.org/10.1038/nmeth1113.

(9) Yu, F.; Haynes, S. E.; Nesvizhskii, A. I. IonQuant Enables Accurate and Sensitive Label-Free Quantification With FDR-Controlled Match-Between-Runs. *Mol. Cell. Proteomics* **2021**, *20*, 100077. https://doi.org/10.1016/j.mcpro.2021.100077.

(10) Mellacheruvu, D.; Wright, Z.; Couzens, A. L.; Lambert, J.-P.; St-Denis, N. A.; Li, T.; Miteva, Y. V.; Hauri, S.; Sardiu, M. E.; Low, T. Y.; Halim, V. A.; Bagshaw, R. D.; Hubner, N. C.; al-Hakim, A.; Bouchard, A.; Faubert, D.; Fermin, D.; Dunham, W. H.; Goudreault, M.; Lin, Z.-Y.; Badillo, B. G.; Pawson, T.; Durocher, D.; Coulombe, B.; Aebersold, R.; Superti-Furga, G.; Colinge, J.; Heck, A. J. R.; Choi, H.; Gstaiger, M.; Mohammed, S.; Cristea, I. M.; Bennett, K. L.; Washburn, M. P.; Raught, B.; Ewing, R. M.; Gingras, A.-C.; Nesvizhskii, A. I. The CRAPome: A Contaminant Repository for Affinity Purification–Mass Spectrometry Data. *Nat. Methods* **2013**, *10* (8), 730–736. https://doi.org/10.1038/nmeth.2557.

(11) Li, K.; Vaudel, M.; Zhang, B.; Ren, Y.; Wen, B. PDV: An Integrative Proteomics Data Viewer. *Bioinformatics* **2019**, *35* (7), 1249–1251. https://doi.org/10.1093/bioinformatics/bty770.

(12) Abramson, J.; Adler, J.; Dunger, J.; Evans, R.; Green, T.; Pritzel, A.; Ronneberger, O.; Willmore, L.; Ballard, A. J.; Bambrick, J.; Bodenstein, S. W.; Evans, D. A.; Hung, C.-C.; O'Neill, M.; Reiman, D.; Tunyasuvunakool, K.; Wu, Z.; Žemgulytė, A.; Arvaniti, E.; Beattie, C.; Bertolli, O.; Bridgland, A.; Cherepanov, A.; Congreve, M.; Cowen-Rivers, A. I.; Cowie, A.; Figurnov, M.; Fuchs, F. B.; Gladman, H.; Jain, R.; Khan, Y. A.; Low, C. M. R.; Perlin, K.; Potapenko, A.; Savy, P.; Singh, S.; Stecula, A.; Thillaisundaram, A.; Tong, C.; Yakneen, S.; Zhong, E. D.; Zielinski, M.; Žídek, A.; Bapst, V.; Kohli, P.; Jaderberg, M.; Hassabis, D.; Jumper, J. M. Accurate Structure Prediction of Biomolecular Interactions with AlphaFold 3. *Nature* **2024**, *630* (8016), 493–500. https://doi.org/10.1038/s41586-024-07487-w.

(13) Meng, E. C.; Goddard, T. D.; Pettersen, E. F.; Couch, G. S.; Pearson, Z. J.; Morris, J. H.; Ferrin, T. E. UCSF CHIMERAX : Tools for Structure Building and Analysis. *Protein Sci.* **2023**, *32* (11), e4792. https://doi.org/10.1002/pro.4792.
